# The Role of Glia in the Peripheral and Central Auditory System Following Noise Overexposure: Contribution of TNF-α and IL-1β to the Pathogenesis of Hearing Loss

**DOI:** 10.3389/fnana.2017.00009

**Published:** 2017-02-23

**Authors:** Verónica Fuentes-Santamaría, Juan Carlos Alvarado, Pedro Melgar-Rojas, María C. Gabaldón-Ull, Josef M. Miller, José M. Juiz

**Affiliations:** ^1^Instituto de Investigación en Discapacidades NeurológicasAlbacete, Spain; ^2^Facultad de Medicina, Universidad de Castilla-La ManchaAlbacete, Spain; ^3^Center for Hearing and Communication Research and Department of Clinical Neuroscience, Karolinska InstitutetStockholm, Sweden; ^4^Kresge Hearing Research Institute, University of MichiganAnn Arbor, MI, USA

**Keywords:** inflammation, cochlear nucleus, inner ear, cytokines, auditory system

## Abstract

Repeated noise exposure induces inflammation and cellular adaptations in the peripheral and central auditory system resulting in pathophysiology of hearing loss. In this study, we analyzed the mechanisms by which noise-induced inflammatory-related events in the cochlea activate glial-mediated cellular responses in the cochlear nucleus (CN), the first relay station of the auditory pathway. The auditory function, glial activation, modifications in gene expression and protein levels of inflammatory mediators and ultrastructural changes in glial-neuronal interactions were assessed in rats exposed to broadband noise (0.5–32 kHz, 118 dB SPL) for 4 h/day during 4 consecutive days to induce long-lasting hearing damage. Noise-exposed rats developed a permanent threshold shift which was associated with hair cell loss and reactive glia. Noise-induced microglial activation peaked in the cochlea between 1 and 10D post-lesion; their activation in the CN was more prolonged reaching maximum levels at 30D post-exposure. RT-PCR analyses of inflammatory-related genes expression in the cochlea demonstrated significant increases in the mRNA expression levels of pro- and anti-inflammatory cytokines, inducible nitric oxide synthase, intercellular adhesion molecule and tissue inhibitor of metalloproteinase-1 at 1 and 10D post-exposure. In noise-exposed cochleae, interleukin-1β (IL-1β), and tumor necrosis factor α (TNF-α) were upregulated by reactive microglia, fibrocytes, and neurons at all time points examined. In the CN, however, neurons were the sole source of these cytokines. These observations suggest that noise exposure causes peripheral and central inflammatory reactions in which TNF-α and IL-1β are implicated in regulating the initiation and progression of noise-induced hearing loss.

## Introduction

Prolonged exposure to excessive noise causes permanent alterations in peripheral and central components of the auditory pathway that may induce progressive sensorineural hearing loss (Saunders et al., 1991; Le Prell et al., 2003, 2007; Abi-Hachem et al., 2010; Fetoni et al., 2015). High-level noise damages the auditory receptor, decreases cochlear blood supply, affects peripheral synapses, and survival of spiral ganglion (SG) neurons (SGN) and the integrity of fibrocytes in the spiral limbus (SLB) and the spiral ligament (SL; Bohne and Harding, 2000; Nordmann et al., 2000; Ou et al., 2000; Wang et al., 2002; Hirose and Liberman, 2003; Park et al., 2013; Kujawa and Liberman, 2015; Sanz et al., 2015), resulting in acute and long-term remodeling of central auditory connections (Bilak et al., 1997; Morest et al., 1998; Muly, 2002; Kim et al., 2004; Muly et al., 2004). One 2 h exposure to harmful noise (118–124 dB) is sufficient to induce in the rodent's inner ear an early upregulation of proinflammatory cytokines/chemokines and intercellular mediators along with downregulation of matrix metalloproteinases and infiltration of macrophages during the acute phase of inflammation (Hirose et al., 2005; Fujioka et al., 2006; Tornabene et al., 2006; Hu et al., 2012).

From animal studies of noise-induced hearing loss (NIHL) it has been postulated that fibrocytes in the cochlear lateral wall are major modulators of acute inflammation processes as they are primarily affected by noise and can produce inflammatory signaling molecules (Tan et al., 2013; Okano, 2014). Previous *in vitro* studies demonstrating that cultured murine SL fibrocytes respond to pro-inflammatory triggers by secreting soluble mediators associated with the immune response support this view (Yoshida et al., 1999; Ichimiya et al., 2000, 2003; Moriyama et al., 2007). Thus, fibrocytes could release proinflammatory cytokines such as interleukin (IL)-1β, tumor necrosis factor (TNF)-α, inducible nitric oxide (NO), monocyte chemoattractant protein-1 (MCP-1), macrophage inflammatory protein (MIP), intercellular adhesion molecule-1 (ICAM-1), and vascular endothelial growth factor (VEGF), which collectively, mediate intercellular communication in the immune system and contribute to prolong inflammation. In addition to fibrocytes and infiltrating leukocytes, other cell types such as microglial-like cells (MLC) may be crucial in triggering peripheral inflammatory events (Okano et al., 2008), as modifications in their expression and/or signaling regulate degenerative and/or regenerative events.

MLC are bone marrow-derived cells, which act as resident tissue macrophages in the inner ear under physiological conditions (Bhave et al., 1998; Lang et al., 2006; Okano et al., 2008). Upregulation of MLC in response to local cochlear damage induced by aminoglycoside ototoxicity or noise exposure has been reported previously in rodents and birds (Bhave et al., 1998; Wang and Li, 2000; Ladrech et al., 2007; Sun et al., 2015). While the role that MLC play in the inner ear remains elusive, a prevailing view is that microglial over-activation is an early detrimental event that precedes cell damage. Current evidence supporting this view is mixed. Thus, inhibition of MLC activation minimizes hair cell loss and improves hearing function following cochlear damage (Sun et al., 2015); while ultrastructural observations have demonstrated that MLC can replace degenerating OHC in the neomycin-damaged organ of Corti, which indicates that efficient cell debris clearance by microglia is beneficial to promote regeneration in the affected cochlea (Wang and Li, 2000).

In the central auditory pathway reactive microglia and astrocytes seem to play a more active role following injury as they are far more numerous than in the peripheral system and act jointly to regulate brain inflammation and minimize brain injury (Bruce-Keller, 1999; Mrak and Griffin, 2005; Cullheim and Thams, 2007; Hanisch and Kettenmann, 2007; Skaper, 2007). Treatment of microglia with astrocyte culture-conditioned media reveals that soluble factor(s) released from astrocytes modulate the production of reactive oxygen and nitrogen species (ROS/RNS) by microglia, leading to the suggestions that astrocyte-microglial interactions contribute to regulation of brain inflammation (Min et al., 2006; Shih et al., 2006; Kim et al., 2010; Luo and Chen, 2012). In addition, cytokines and downstream mediators of the inflammatory cascade have modulatory effects on neuronal excitability, acting directly through receptor binding or indirectly by stimulating the release of other neuroactive molecules (Rasmussen et al., 2007; Vezzani et al., 2008; Madinier et al., 2009).

In the cochlear nucleus (CN), immunohistochemical studies have demonstrated that damage to the inner ear, by cochlear ablation or noise-exposure, induces an activated microglial phenotype (Campos-Torres et al., 1999; Janz and Illing, 2014; Baizer et al., 2015) and rapid and long-lasting interactions between microglial cells and injured auditory neurons (Fuentes-Santamaría et al., 2012). Such neuronal-microglial signaling is critical to intercellular communication and facilitates extracellular chemical signaling. Deprivation of afferent activity also leads to protracted and persistent reactive gliosis in the CN (Lurie and Rubel, 1994; de Waele et al., 1996; Fredrich et al., 2013; Fuentes-Santamaría et al., 2013) which may contribute to synaptic regrowth or repair following deafferentation (Benson et al., 1997; Muly, 2002; Hildebrandt et al., 2011). Although microglial cells and astrocytes release a variety of cytokines and growth factors upon activation (Mason et al., 2001; Hanisch, 2002; Fogal and Hewett, 2008), the upregulation of proinflammatory molecules and growth factors in the CN after bilateral cochlear ablation in rats, occurred only within neurons and not in reactive astrocytes or microglia (Fuentes-Santamaría et al., 2013). These observations indicate that additional synthesis of these soluble mediators by glial cells might not be essential to restore central auditory processing in response to neurosensorial deafness.

In this study, adult Wistar rats were exposed to broadband noise (0.5–32 kHz, 118 dB SPL) for 4 h/day during 4 consecutive days to induce permanent auditory damage. The spatiotemporal expression profile and activation of glial cells as well as the gene expression profile of proinflammatory cytokines, cell adhesion molecules, neurotoxic mediators, and metalloproteinases were assessed in the inflamed cochlea. Specifically, the sources of the inflammatory mediators, TNFα, and IL-1β, which are crucial in triggering inflammatory gene cascades and modulating neuronal function in injured tissue, were evaluated in the noise-exposed cochlea and in the anteroventral cochlear nucleus (AVCN). In addition, glial-neuronal interactions were evaluated in the CN using electron microscopy. These results suggest that following noise overexposure there is a complex interplay between peripheral and central signaling events involving glial cell responses and release of newly synthesized neuroactive molecules such as TNFα and IL-1β, which might be essential to restore glial-neuronal signaling.

## Materials and methods

### Animals

Adult Wistar rats (*n* = 48; 3 months, female, Charles River Laboratories, Barcelona, Spain) were maintained on a 12 h light/dark cycle with food and water *ad libitum* at the Universidad of Castilla-La Mancha Animal house (Albacete, Spain). The procedures involving the use and care of the animals were approved by the corresponding Institutional Animal Care and Use Committee (Permit Number: PR-2013-02-03). These protocols were in accordance with the guidelines of the European Communities Council (Directive 2010/63/EU) and current national legislation (R.D. 53/2013; Law 32/2007).

### Auditory function evaluation

#### Auditory brainstem responses (ABR)

ABR testing was conducted as described elsewhere (Alvarado et al., 2012). Only animal subjects that exhibited a normal hearing function were included in this study. Testing took place in a sound-attenuating, electrically shielded booth (EYMASA/INCOTRON S.L., Barcelona, Spain) placed inside a sound-attenuating room. Rats were anesthetized with isoflurane (1 L/min O2 flow rate) at 4% for induction and 1.5–2% for maintenance and during recordings, their temperature was monitored with a rectal probe and maintained at 37.5 ± 1°C using a non-electrical heating pad. Subdermal needle electrodes (Rochester Electro-Medical, Tampa, FL, USA) were positioned at the vertex (non-inverting) and in the right (inverting) and the left (ground) mastoids. Sound stimulation and recordings were performed using a BioSig System III (Tucker-Davis Technologies, Alachua, FL, USA). The acoustic stimuli were pure tone bursts sounds (5 ms rise/fall time without a plateau with a cos2 envelope delivered at 20/s) at seven different frequencies (0.5, 1, 2, 4, 8, 16, and 32 kHz). The sounds were generated digitally by the SigGenRP software (Tucker-Davis Technologies, Alachua, FL, USA) and the RX6 Piranha Multifunction Processor (Tucker-Davis Technologies, Alachua, FL, USA), and were delivered into the right ear using an EDC1 electrostatic speaker driver (Tucker-Davis Technologies) through an EC-1 electrostatic speaker (Tucker-Davis Technologies). Calibration was performed prior to the experiments using SigCal software (Tucker-Davis Technologies) and an ER-10B+ low noise microphone system (Etymotic Research Inc., Elk, Groove, IL, USA). Evoked responses were filtered (0.3–3.0 kHz), averaged (500 waveforms) stored for offline analysis. The ABR recordings were evaluated the day before sound stimulation and at the end of each survival time.

#### Noise exposure protocol

This noise stimulation paradigm consisted of exposure to broadband noise at 118 dB SPL, for 4 h a day during 4 consecutive days. The sound was delivered inside of a methacrylate reverberating chamber with 60 × 70 × 40 (length × width × height) cm with tilted and non-parallel walls with the purpose to avoid standing waves and ensure a more homogeneous sound field. The chamber was placed into a double wall sound-attenuating booth located inside a sound-attenuating room. During the procedure, animals were awake, move freely around the chamber and had access to water. Noise-exposed rats of each group were either perfused or sacrificed at 1 (1D), 10 (10D), and 30 (30D) days post-exposure (PE).

#### Data analysis

The assessment of the “auditory threshold” was conducted by recording evoked responses in 5 dB steps descending from 80 dB sound pressure level (SPL). For each of the frequencies evaluated, the auditory threshold was defined as the stimulus intensity that evoked waveforms with a peak-to-peak voltage >2 standard deviations (*SD*) from the background activity measured before the stimulus onset (Cediel et al., 2006; Garcia-Pino et al., 2009; Alvarado et al., 2012, 2014; Fuentes-Santamaría et al., 2012). The maximum intensity level was 80 dB (Gourévitch et al., 2009; Alvarado et al., 2012, 2014; Fuentes-Santamaría et al., 2013; Melgar-Rojas et al., 2015) to reduce the possibility of inducing acoustic trauma in unexposed animals and additional overstimulation in noise-exposed rats during the recordings. Following the noise stimulation protocol, if no evoked responses were obtained during the recording at 80 dB, the auditory thresholds were set at that value for statistical analysis and comparisons (Subramaniam et al., 1992; Trowe et al., 2008; Alvarado et al., 2012, 2014; Fuentes-Santamaría et al., 2014; Melgar-Rojas et al., 2015). “*The threshold shift*” was defined as the difference between the auditory thresholds following overstimulation, minus the auditory thresholds in the unexposed condition, for each animal at each of the frequencies tested (Alvarado et al., 2012, 2014; Fuentes-Santamaría et al., 2012, 2013, 2014; Melgar-Rojas et al., 2015).

### Cochlear immunohistochemistry

Unexposed (*n* = 5) and noise-exposed (*n* = 15) rats were anesthetized with an intraperitoneal injection of pentobarbital (200 mg/Kg) and transcardially perfused with 0.9% saline wash followed by 4% paraformaldehyde solution in 0.1 M phosphate buffer (PB, pH 7.3). Left cochleae were removed from the temporal bone and decalcified in 50% RDO rapid decalcifier solution (Apex Engineering Products Corporation, Illinois, USA) for 2 h. Then, they were embedded in a solution of 15% sucrose and 10% gelatin, frozen at −70°C by immersion in 2-propanol/dry ice bath and sectioned at 20 μm on a cryostat. After several rinses in phosphate-buffered saline (PBS) containing 0.2% Triton X-100 (Tx), cochlear sections were blocked for 1 h in PBS-Tx (0.2%) containing 10% normal goat serum (NGS) and incubated overnight in a humid chamber at 4°C with ionized calcium-binding adaptor 1 (Iba1) primary antibody (Supplementary Table 1) diluted in a solution containing PBS-Tx (0.2%), pH 7.4. The following day, after four 15 min rinses in PBS-Tx (0.2%), sections were incubated for 2 h in biotinylated goat anti-rabbit (1:200, Vector Laboratories, Burlingame, CA, USA) secondary antibody and for 1 h in the avidin-biotin -peroxidase complex solution (ABC) to visualize the bound antibody. The DAB-treated sections were counterstained with cresyl violet staining and coverslipped using Cytoseal (Stephens Scientific). Immunofluorescent sections were processed as above, but incubated in goat anti-rabbit conjugated to Alexa 594 (Molecular Probes, Eugene, OR, USA) and counterstained with DAPI.

### Cochlear whole-mount surface preparations

The right cochleae from the same rats were processed for whole-mount surface preparations (Melgar-Rojas et al., 2015). After cochlear decalcification, the sensory epitheliums were collected, and microdissected into individual turns that were incubated for 24 h at 4°C in prestin antibody (Supplementary Table 1) diluted in PBS-Tx (0.2%). After several rinses in PBS-Tx (0.2%), each organ of Corti was incubated in donkey anti-goat conjugated to Alexa 594 secondary antibody for 2 h, rinsed in PBS, mounted on glass slides, counterstained with DAPI nuclear staining and coverslipped. Immunofluorescence was examined with a laser scanning confocal microscope (LSM 710; Zeiss, Germany) with excitation laser lines at 405 and 594 nm. Z-stack series of 3–5 μm thickness were acquired as images of 1024 × 1024 pixels recorded at intervals of 0.5 μm with a 40X Plan Apo oil-immersion objective (1.4 NA).

### Quantification of surviving OHC

Cells were counted in segments of ~250 μm in length each along the basilar membrane by using the public domain image analysis software Scion Image for Windows (version beta 4.0.2; developed by Scion Corp). Dark spots and/or the phalangeal scars of supporting cells in the spaces previously occupied by OHC were used as criteria to assess sensory cell loss (Hu and Henderson, 1997; Minami et al., 2007; Fetoni et al., 2013). The results were expressed as percentage of remaining OHC compared to control condition, over the length of the basilar membrane (Viberg and Canlon, 2004; Fetoni et al., 2013). Image acquisition was performed with a laser scanning confocal microscope (LSM 710; Zeiss, Germany). By using the ZEN 2009 Light Edition software (Zeiss, Germany), the images of each dye were captured sequentially with a 40X Plan Apo oil-immersion objective (1.4 NA), merged and saved as TIFF files.

### Cochlear dissection, RNA extraction, and cDNA synthesis

Tissue collection from control (*n* = 4) and experimental animals (*n* = 12), RNA extraction and cDNA synthesis were performed as previously described (Melgar-Rojas et al., 2015). Briefly, the whole cochlea was dissected from the temporal bone of euthanized animals, quickly frozen in dry ice and stored at −80°C. Total RNA was extracted from frozen samples by following the TRIzol (Invitrogen) standard protocol and purity. Integrity and concentration of each RNA sample was measured by electrophotometric (Nanodrop ND-1000, Thermo Scientific) and electrophoretic methods (agarose gels). The synthesis of single-strand cDNAs was performed by using the RevertAid First Strand cDNA Synthesis Kit (Thermo Scientific) from 1 μg of RNA. The reactions were carried out in a DNA Engine® Peltier Thermal Cycler (BioRad) and in a final volume of 20 μl. Reaction conditions were as previously published (Melgar-Rojas et al., 2015). After the reaction, cDNAs were diluted 1:10 in sterile H_2_O MilliQ.

### Primer design and reverse transcriptase-quantitative polymerase chain reaction (RT-qPCR)

RT-qPCRs were performed using specific primer pairs for amplifying transcripts of genes of interest (GOIs) that were designed using the Primer3 Plus software (http://www.bioinformatics.nl/cgi–bin/primer3plus/primer3plus.cgi/; Supplementary Table 2). Gene specificities were tested by BLAST analysis (NCBI) and every primer was matched against its genomic sequence (Ensembl Data Base) to check its genomic location and to avoid false-positive amplification in the case of genomic DNA contamination.

The RT-qPCR amplification was performed in a One Step Plus Real-Time PCR System machine (Applied Biosystems) using Fast SYBR Green Master Mix (Applied Biosystems) as dye reagent. The reaction mix per well-consisted of 2.8 μl of sterile H_2_O MilliQ, 0.1 μl of forward and reverse primer (final concentration of 100 nM), 5 μl of Fast SYBR Green Master Mix and 2 μl of 10-fold diluted cDNA. The RT-qPCR program and the melting curve generation were carried out as previously described (Melgar-Rojas et al., 2015) and confirmed that the primers amplified only one specific PCR product. The amplification efficiencies (*E*-values) and correlation coefficients (*R*^2^-values) of each primer pair were obtained also as previously reported (Melgar-Rojas et al., 2015; Supplementary Table 2). Specifically, *E*-values were calculated by the following formula: Efficiency (%) = [–1 + 10(– 1/slope)] × 100. All the RT-qPCR plates included non-template controls (NTC) which generated *Cq* > 35. The experiments were performed technically in triplicate and biologically in quadruplicate.

Quantification of expression (fold change) from the Cq data was calculate using Step One Software v2.3 (Applied Biosystems) and following the ΔΔCq method (Schmittgen and Livak, 2008). Briefly, the expression level of GOIs were first normalized to the average level (the geometric mean as recommended in Vandesompele et al., 2002) of *Hprt1* and *Tbp* which were the most stably expressed along time in the rat cochlea in response to noise exposure. After obtaining the ΔCq, the ΔΔCq of each GOI was calculated as: ΔCq (noise-exposed group) −ΔCq (control group). Relative expression (fold change) was calculated as: 2^−ΔΔCq^. All RT-qPCR experiments were compliant with the MIQE guidelines (Bustin et al., 2009).

### CN immunohistochemistry

Brains obtained from the same animals used for cochlear histology were removed from the cranium, and sucrose-cryoprotected for 48 h. Frozen sections 40 μm thick were cut on a sliding microtome in the coronal plane. The immunohistochemistry procedure followed was the same as indicated earlier for cochlear sections excepting that CN sections were also incubated with GFAP primary antibody (Supplementary Table 1). To evaluate the specificity of the immunohistochemistry detection system the following sets of control experiments were assessed: (1) omission of the primary antibody by replacement with TBS-BSA; (2) omission of secondary antibodies; and (3) omission of ABC reagent. Under these conditions, no immunostaining was detected. Boundaries between CN subdivisions were defined in accordance with previous studies in rats describing the distribution and cytoarchitecture of auditory neurons in these cellular domains (Mugnaini et al., 1980; Cant and Benson, 2003).

### Double immunofluorescence labeling

Adjacent sections from these same animals were used for double-labeling studies. Both cochlear and CN sections were rinsed several times in 0.2% TBS-Tx and blocked for 1 h in the same buffer solution containing 10% normal goat serum. Cochlear sections were incubated overnight in a solution containing either Iba1 or TNF-α/Iba1, or IL-1β-/Iba1 or calretinin (CR)/Iba1 primary antibodies. Meanwhile, brain sections were double-labeled with either TNF-α-/Iba1, or TNF-α/glial fibrillary acidic protein (GFAP) or IL-1β/Iba1 or IL-1β/GFAP or triple-labeled with NeuN/Iba1/GFAP primary antibodies (Supplementary Table 1). After several rinses in TBS-Tx 0.2%, sections were incubated in the corresponding cocktail of fluorescently labeled secondary antibodies [1:200, donkey anti-goat conjugated to Alexa 488 and donkey anti-rabbit conjugated to Alexa 594 for TNF-α/Iba1, IL-1β-/Iba1, calretinin (CR)/Iba1, and goat anti-rabbit conjugated to Alexa 488 and goat anti-mouse conjugated to Alexa 594 for NeuN/GFAP; Molecular Probes, Eugene, OR, USA] for 2h at room temperature and coverslipped with DAPI. Fluorescent sections were examined with confocal microscopy (Zeiss LSM710 multichannel laser scanning confocal imaging system).

### Data analysis

#### Quantification of the immunostaining

Iba1 immunostaining was examined using a 40× objective on a Nikon Eclipse 80i photomicroscope (Nikon Instruments Europe B.V.) and images were captured using a DXM 1200C digital camera (Nikon Instruments Europe B.V.) that was attached to the microscope. The color images of each field were digitized, and the resultant 8-bit red channel images, containing a grayscale of pixel intensities from 0 (white) to 255 (black), were used for analysis. In the cochlea, the analysis was performed on equally spaced (80 μm apart) mid-modiolar sections. Two microscopic 60× fields (dorsal and ventral) per section were sampled in the spiral ganglion and the spiral ligament. In the AVCN, the immunostaining was measured on equally spaced (160 μm apart) coronal sections. For each section, three microscopic 60× fields (dorsal, middle, and ventral) extending through the rostrocaudal dimension of the nucleus were selected, photographed and analyzed. As described elsewhere (Fuentes-Santamaría et al., 2003, 2005, 2007a,b; Alvarado et al., 2004, 2009a), the immunostaining was evaluated by using the public domain image analysis software Scion Image for Windows (version beta 4.0.2; developed by Scion Corp). For suitable comparisons of the staining across samples, a macro was used to process and analyze the captured images (Alvarado et al., 2004). Then, the images were normalized, the threshold level was set at two *SD* above the mean gray level of the field, and the immunostained elements above this threshold were identified as labeled.

#### Immunostained indexes and percentage of variation of the immunostaining

For quantitative analysis of the immunostaining, the following indexes were calculated: (1) *the mean gray level of the immunostaining*, which was used as an indirect indicator of protein levels within cells and (2) *the percentage of variation of the immunostaining*, which was calculated by using an enhancement index, according to the following formula (Meredith and Stein, 1983; Alvarado et al., 2007a,b, 2009b, 2016).

$$\begin{array}{l} \text{\% of variation}=\left[\left(\text{IEC }-\text{ IUC}\right)/\left(\text{IUC}\right)\right]\times 100 \end{array}$$

Where IEC and IUC represent the mean gray level in the noise-exposed and unexposed conditions; respectively.

#### Quantification of glial-neuronal interactions

For quantification of presumptive interactions among neurons, astrocytes, and microglia, four sections (43672.47 μm^2^) from five animals were selected from each group. For each section, three microscopic fields extending through the rostrocaudal dimension of the nucleus were sampled using a 40X objective. The colocalization area (NeuN/Iba1 or NeuN/GFAP) was estimated from representative *z*- stack confocal microscopy images by using the public domain image analysis software Scion Image for Windows (version beta 4.0.2; developed by Scion Corp), as previously described (Fuentes-Santamaria et al., 2008). The immunostained profiles above the threshold were identified and the resultant images were converted into binary. To determine the degree of colocalization, the binary images corresponding to a given antibody were pseudocolored (green for Iba1 or red for GFAP) and then merged with the other pseudocolored image (blue for NeuN). The resultant image was used to quantified the overlapped area (putative region of interaction). The *percentage of variation* was calculated as above mentioned:

$$\begin{array}{l} \text{\% of variation}=\left[\left(\text{AEC }-\text{ AUC}\right)/\left(\text{AUC}\right)\right]\times 100 \end{array}$$

Where AEC and AUC represent the immunostained colocalized area in the noise-exposed and unexposed conditions, respectively.

### Pre-embedding electron microscopy

After intracardial perfusion with 0.9% saline wash followed by a fixative solution of 4% paraformaldehyde and 0.5% glutaraldehyde (0.1 M PB, pH 7.3), unexposed (*n* = 3) and noise-exposed (*n* = 9) brains were removed, and cut at 70 μm on a vibratome. After several rinses in PBS-Tx (0.2%), transverse sections were processed for Iba1 or GFAP immunohistochemistry as previously described with the exception that no detergent was used in any step after the initial permeabilization. Peroxidase activity was visualized with a nickel-intensified DAB reaction in buffer solution to produce a black reaction product. CN sections were postfixed with osmium tetraoxide (1% in 0.1 M PB) for 1 h, block-stained with 1% uranyl acetate for 30 min, dehydrated in graded series of ethanol and embedded in Durcupan (Fluka) resin (Fuentes-Santamaría et al., 2014). Ultrathin sections (~75 nm) in the silver-gold range were cut on an ultramicrotome (Reichert Ultracut E, Leica, Austria), collected on 200-mesh copper grids and examined with a Jeol-1010 electron microscope.

### Preparation of figures and statistical analysis

Photoshop (Adobe v5.5) and Canvas (Deneba v6.0) software packages were used to adjust the size, brightness and contrast of the images for the preparation of figures. All data are expressed as means ± SEM. The measurements of the ABRs parameters were performed at 80 dB SPL unless otherwise indicated. One-way analysis of variance (ANOVA) with Scheffé *post-hoc* analysis as necessary was used for comparisons between groups. A *p* < 0.05 was considered statistically significant.

## Results

### Physiological assessment of noise-induced cochlear damage

**Figure 1:**
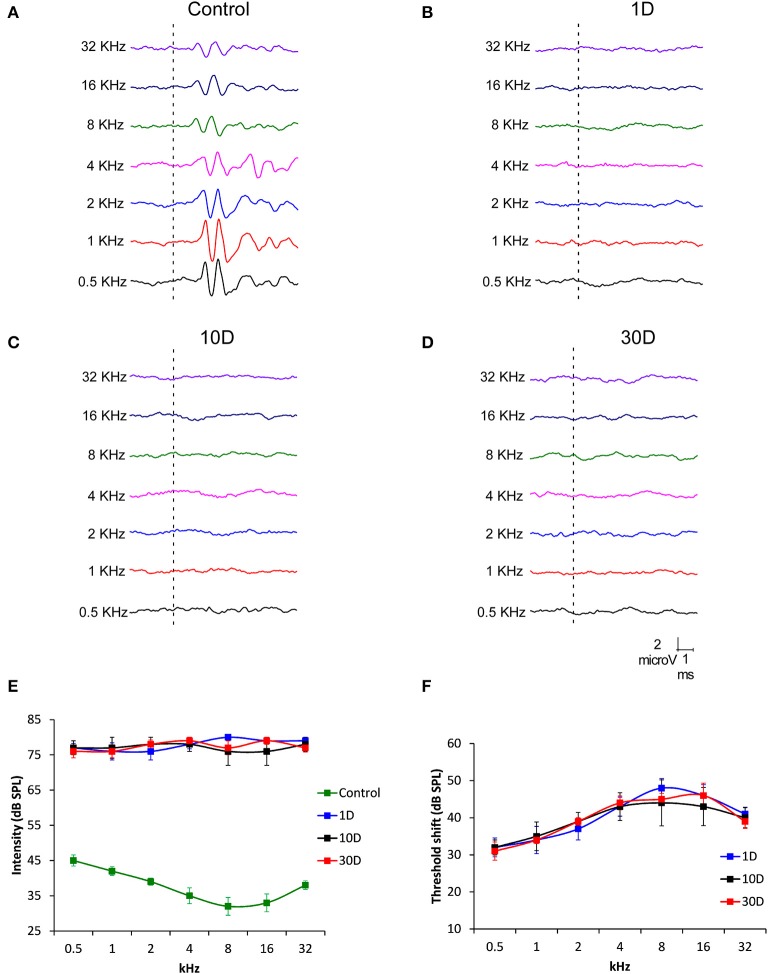
**Line graphs showing ABR recordings (A–D) in control (***n*** = 12) and noise-exposed (***n*** = 36) rats at 80 dB SPL for frequencies between 0.5 and 32 kHz**. When compared to the ABR pattern of unexposed rats **(A)**, noise exposure (118 dB SPL) for 4 h/day during 4 consecutive days resulted in an almost complete absence of the typical ABR waves at all frequencies and time points assessed following the exposure **(B–D)**. Progression of the auditory thresholds **(E,F)** indicated that noise-exposed rats at all survival time**s** had higher values than those observed in unexposed animals **(E)**. The threshold shift values fluctuated between 32 and 42 dB **(F)**. Recordings in **(A–D)** are represented at 80 dB SPL.

To determine hearing dysfunction following exposure to a high level of noise, ABR recordings were conducted in noise-exposed rats at 1, 10, and 30D post-exposure. When compared to unexposed rats which had a distinctive pattern of 4–5 waves after the stimulus onset (Figure 1A), the recordings in exposed rats showed an almost complete elimination of the waves at all frequencies and time points evaluated (Figures 1B–D). Regarding the absolute hearing threshold in experimental rats, they were higher than in unexposed animals and very similar across frequencies (Figure 1E). The mean thresholds in noise-exposed animals were above 75 dB SPL while in unexposed rats the average thresholds were higher at the lowest frequencies, reduced at medium frequencies and augmented again at the highest frequencies (Figure 1E). At all post-exposure time points evaluated, the threshold shift ranged from 32 to 42 dB and showed no sign of recovery between day 1 and 30 following the exposure, indicating that experimental animals had permanent hearing loss at all frequencies in response to repeated noise exposure (Figure 1F).

### Histological assessment of noise-induced cochlear damage

#### Loss of OHC and upregulation of prestin

**Figure 2:**
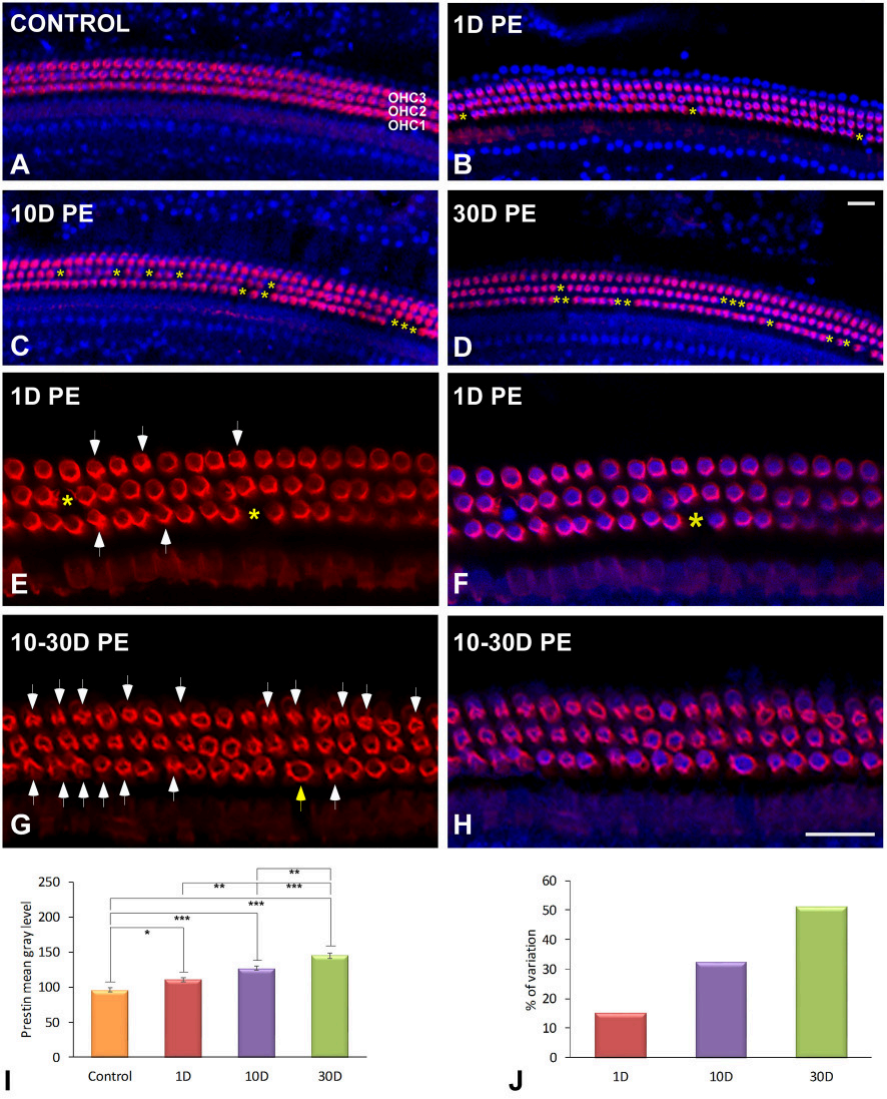
**Surface preparation images illustrating double-labeling of prestin (red) and DAPI (blue) in the middle cochlear turn in control (A) and noise-exposed (B–H) rats**. The survival of OHC was significantly decreased with longer time points post-exposure. Loss of OHC are indicated by yellow asterisks. Arrows in **(E,G)** indicate disrupted OHC stained with prestin protein. The yellow arrow in **(G)** indicates a swollen nucleus. Quantification of the mean gray levels indicated noise-induced increases in prestin staining when compared to control cochleae **(I)**. The percentage of variation of the immunostaining in noise-exposed (*n* = 5 for each group) animals relative to control (*n* = 5) is shown in **(J)**. The time of sacrifice following exposure (PE) is indicated in the upper left of each image. Significant differences in prestin immunostaining among animal groups **(I)** are indicated by asterisks (^*^p < 0.05; ^**^p < 0.01, ^***^p < 0.001). Scale bars: 20 μm in **(D,H)**.

Evidence of OHC damage was assessed by determining the percentage of cell loss and the expression of prestin protein, which is highly sensitive to noise overstimulation and essential for OHC electromotility (Figure 2). When compared to unexposed rats (Figure 2A), there was a high percentage of surviving OHC at 1D post-exposure (Figure 2B; Supplementary Table 3) which was concomitant with a significant increase in prestin staining in the basolateral membrane of OHC (Figures 2E,F; Supplementary Table 4). At later time points after overstimulation, OHC integrity in the first and second rows was mostly compromised, as the proportion of missing OHC (asterisks in Figure 2C; Supplementary Table 3) and prestin levels in the surviving ones progressively increased over time (Supplementary Table 4). On day 30 post-trauma, the majority of prestin-stained OHC were disrupted and/or degraded in all rows indicating a widespread cell damage, consistent with severe cochlear dysfunction (Figures 2G,H; Supplementary Table 4). Note that a few hair cells with swollen nuclei were evident (yellow arrow in Figure 2G). Quantification of the percentage of surviving OHC following noise-exposure demonstrated that cell death was mostly localized in the middle and basal cochlear turns at all survival times after the exposure (Supplementary Table 3). In the apical turn, however, the cell death was only observed at the longest time point studied (Supplementary Table 3). Upregulation of prestin levels in OHC in noise-exposed rats was confirmed by quantifying the mean gray level intensities of the immunostaining (Figure 2I; Supplementary Table 4). The percentage of variation of the immunostaining indicated that these increases ranged from 15.21 to 51.23% (Figure 2J).

### Effects of noise exposure on MLC in the cochlea

**Figure 3:**
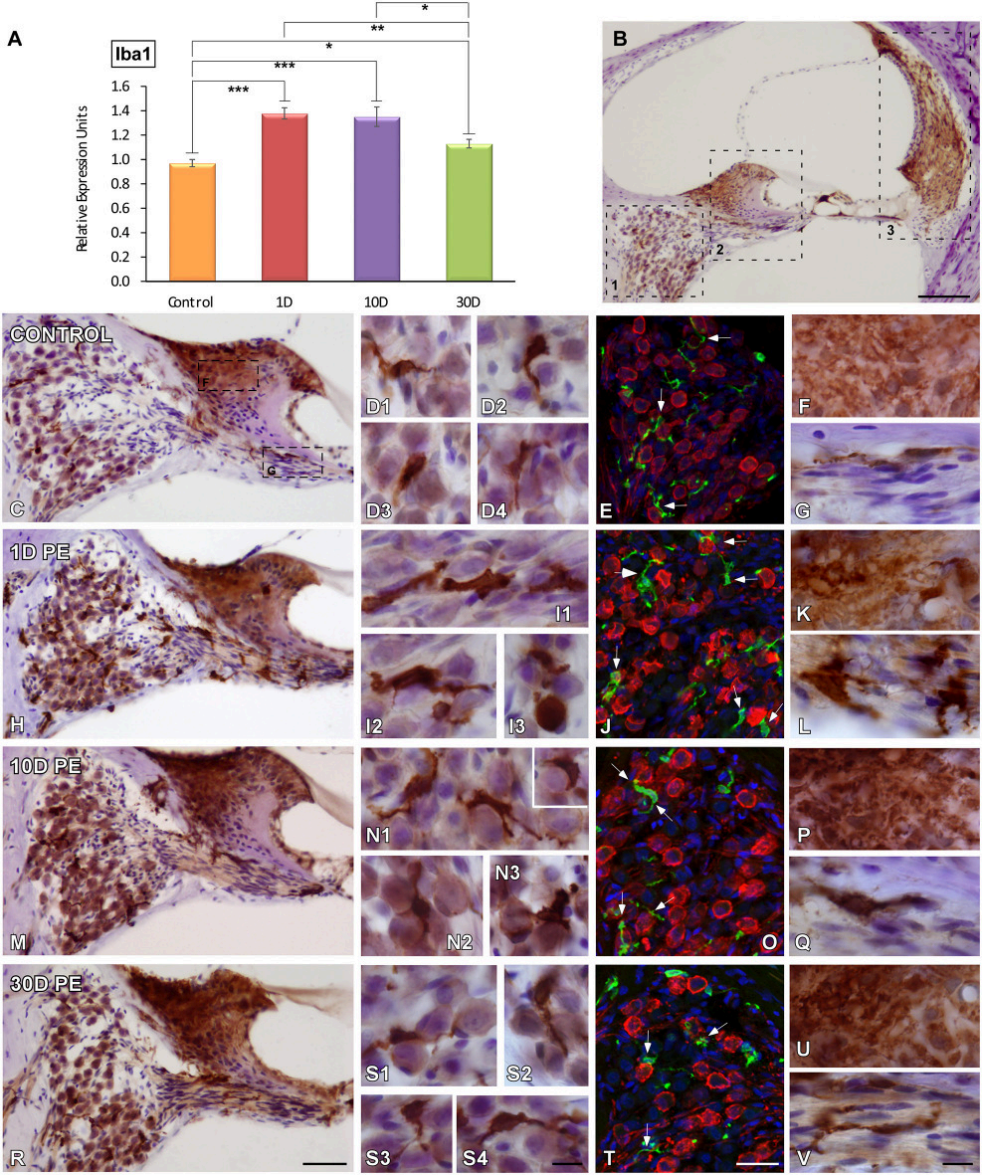
**Iba1 mRNA expression and protein levels in the cochlea of control and noise-exposed rats**. mRNA levels in the noise-exposed cochleae were elevated at all time points post-exposure **(A)**. The time course of MLC activation was examined in noise-vulnerable cochlear regions including the spiral ganglion **(B1)**, the spiral limbus and cochlear nerve **(B2)**, and the spiral ligament **(B3)** at 1, 10, and 30D post-exposure. As compared to control rats **(C–G)**, noise produced increases in MLC activation were observable on day 1, when cells had larger cell bodies and thickened and shortened processes **(H–L)**. Although these cells remained active by 10D post-exposure **(M–Q)**, with longer time points (30D) Iba1-stained cells recovered their ramified appearance **(R–V)**. Confocal images showing close appositions between microglia (green) and SGN (red) are indicated by arrows in (**E,J,O,T;** *n* = 5 for each group). Significant differences in Iba1 mRNA levels among control and noise-exposed animals **(A)** are indicated by asterisks (^*^p < 0.05; ^**^p < 0.01, ^***^p < 0.001). Scale bars: 200 μm in **(B)**; 50 μm in **(R)**; 10 μm in **(S4,V)** and 25 μm in **(T)**.

As microglial cells are very sensitive indicators of alterations in tissue homeostasis and the main contributors to post-lesion adaptive immune responses, their temporal expression and activation in the cochlea were investigated following repetitive sound stimulation by using Iba1, a macrophage/microglia-specific calcium-binding protein. Iba1 mRNA in the cochlea had a maximum expression peak between 1 and 10D post-exposure as compared to the control condition (Figure 3A). On day 30, mRNA levels were still significantly elevated above control values (Figure 3A). Iba1-immunostaining was evaluated in different cochlear structures which are known to be vulnerable to permanent damage caused by noise (Figure 3B).

#### Spiral ganglion

In unexposed rats, scarce Iba1-stained cells with small cell bodies and ramified long processes were distributed among SGN (Figures 3C–E). On day 1 following noise-exposure, reactive MLC were enlarged and intensely stained with thick or no processes that were closely associated with SGN (Figures 3H–J). Note that these microglial cells had an amoeboid phenotype that resemble active macrophages (Figures 3I1–I3). With longer time points post-lesion, the response of microglial activation diminished progressively as reflected by cells with smaller somata and highly branched processes (Figures 3M–O,R–T) when compared to the control condition (Figures 3C–E). In noise-exposed animals, the frequency of SGN-glial contacts decreased over time as did the degree of microglial activation and number of activated microglial cells. In this regard, early activity-dependent increases in CR levels within SGN were correlated with increased microglial activation. These interactions are indicated by arrows in Figures 3E,J,O,T. On days 1 and 10 post-exposure, CR levels were upregulated (Figures 3J,O) in comparison with longer time points (Figure 3T) and unexposed (Figure 3E) animals. Iba1 upregulation within the SG was confirmed by significant increases in the mean gray level of the immunostaining that ranged from 56.12 to 42.88% (Supplementary Table 5).

#### Spiral limbus and cochlear nerve

Similar to what occurs in the SG, there were significant increases in the intensity of Iba1-immunostaining in the SLB and cochlear nerve following noise exposure (Figures 3K,L,P,Q,U,V) when compared to unexposed (Figures 3F,G) rats. At the earliest time point post-exposure, MLC in the cochlear nerve were higher in number and had phenotypic features that resembled that of activated microglia (Figure 3L). This cellular response gradually decreased over time (Figure 3Q) and cells recovered their resting phenotype (Figure 3V).

#### Spiral ligament

**Figure 4:**
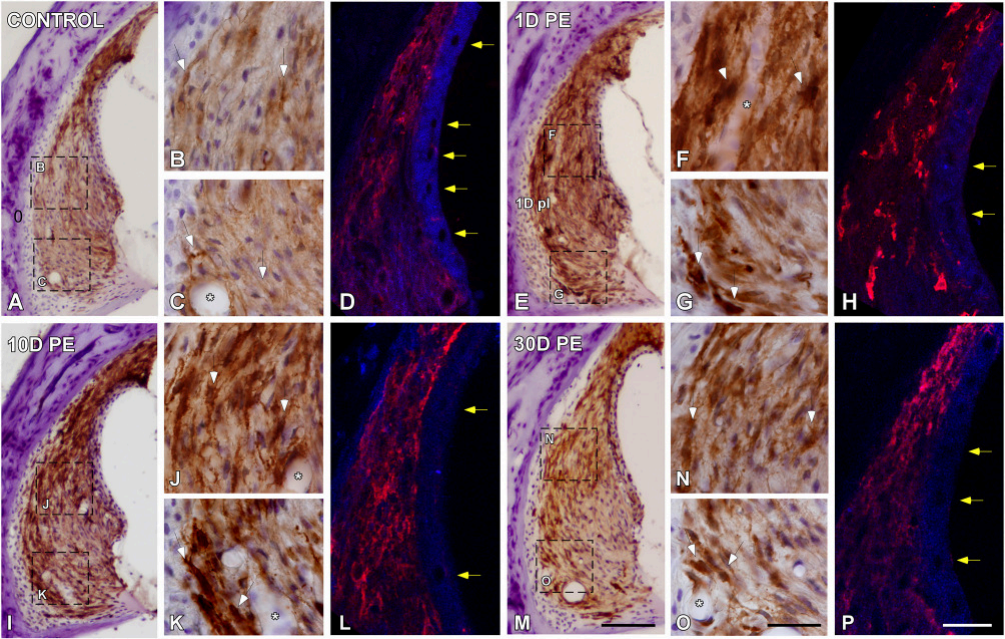
**Digital images showing Iba1-immunostaining in the spiral ligament of control (***n*** = 5) and noise-exposed (***n*** = 5 for each group) rats**. When compared to the control condition **(A–C)**, Iba1-immunostaining at all time points post-exposure evaluated was markedly increase in MLC located in type I and IV fibrocytes regions (white arrows in **E–G,I–K,M–O**). Particularly on day 1, cells were darkly stained and had expanded cell bodies and thickened processes (arrows in **F,G**). Asterisks indicate the association of MLC with blood vessels. Yellow arrows in **(H,L)** point to noise-decreases in blood vessels diameter at 1 and 10D after the exposure in comparison to 30D post-exposure (arrows in **P**) and control (arrows in **D**) rats. Scale bars: 100 μm in M; 20 μm in O; and 25 μm in **(P)**.

In the control condition, Iba-immunostaining was located mostly in the dorsolateral part of the SL, which corresponds to type I fibrocytes region (Figures 4A–D). On day 1, noise-exposure triggered a microglial activation response characterized by an increased number of microglial cells, which were intensely stained, hypertrophic, and with widened short processes (arrows in Figures 4E–H). Immunostaining remained elevated on day 10 (Figures 4I–L) but this enhanced activated phenotype decreased in intensity as function of time (Figures 4M–P). At all time points evaluated, activated microglial cells extended to the area where type IV fibrocytes were distributed (Figures 4E–P). The quantification of Iba1 immunostaining and the percentage of its variation in the SL confirmed the significant increases in the mean gray level (Supplementary Table 5). In the stria vascularis, blood flow modifications were reflected as a decrease in the diameter of blood vessels at earlier time points after noise-stimulation (yellow arrows in Figures 4H,L) and partial recovery on day 30 (yellow arrows in Figures 4P) when compared to the control condition (yellow arrows in Figures 4D). Microglial cells attached to blood vessel are indicated by asterisks in Figure 4.

### Noise-induced upregulation in the expression levels of cytokines, adhesion molecules, tissue inhibitor of metalloproteinases, and iNOS

**Figure 5:**
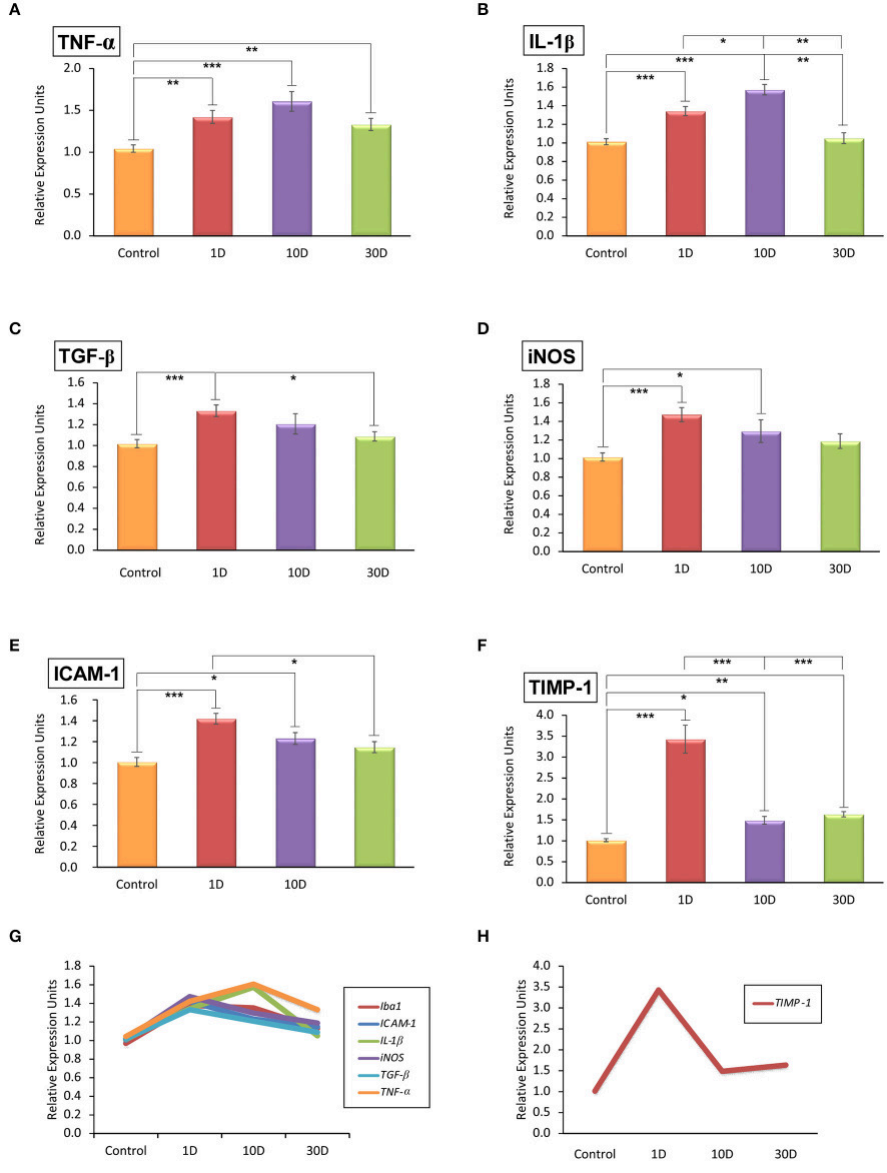
**Quantitative RT-qPCR analyses of inflammatory-related genes expression in the noise-exposed cochlea (***n*** = 4 for each group)**. Significant elevations in the mRNA expression levels of *TNF*-α **(A)**, *IL-1*β **(B)**, *TGF*-β **(C)**, *iNOS* **(D)**, and *ICAM-1* **(E)** were detected after noise-exposure at all time points evaluated. *TIMP-1* expression levels **(F)** were increased transiently on day 1 and quickly downregulated at longer time points post-exposure but without reaching normal levels. The overlapping distribution of genes expressed in response to noise exposure is shown in **(G)** while the particular progression of *TIMP-1* expression is shown in **(H)**. Significant differences among animal groups are indicated by asterisks (^*^p < 0.05; ^**^p < 0.01, ^***^p < 0.001).

To evaluate the inflammatory status in noise-exposed cochleae, the mRNA expression levels of pro-inflammatory cytokines (TNF-α, IL-1β, and TGF-β), intercellular adhesion molecule-1 (ICAM-1), tissue inhibitor of metalloproteinases 1 (TIMP-1), and inducible NO synthase (iNOS) were analyzed by RT-qPCR. The analysis showed that all the inflammatory cytokines and inflammatory factors evaluated were upregulated by 1D and 10D post-stimulation and slowly decreased at longer time points when compared to the control condition (Figure 5; Supplementary Table 6). While the upregulation of TGF-β, iNOS, and ICAM-1 expression peaked at the earliest time point evaluated (Figures 5C–E,G; Supplementary Table 6), that of TNF-α and IL-1β augmented on day 10 after the exposure (Figures 5A,B,G; Supplementary Table 6). Particularly, TIMP-1 expression was transiently upregulated by 1D post-exposure as it decreased markedly on day 10 to remain significantly elevated over control levels on day 30 (Figures 5F,H; Supplementary Table 6).

### TNF-α and IL-1β-expressing cells in the noise-exposed cochlea

**Figure 6:**
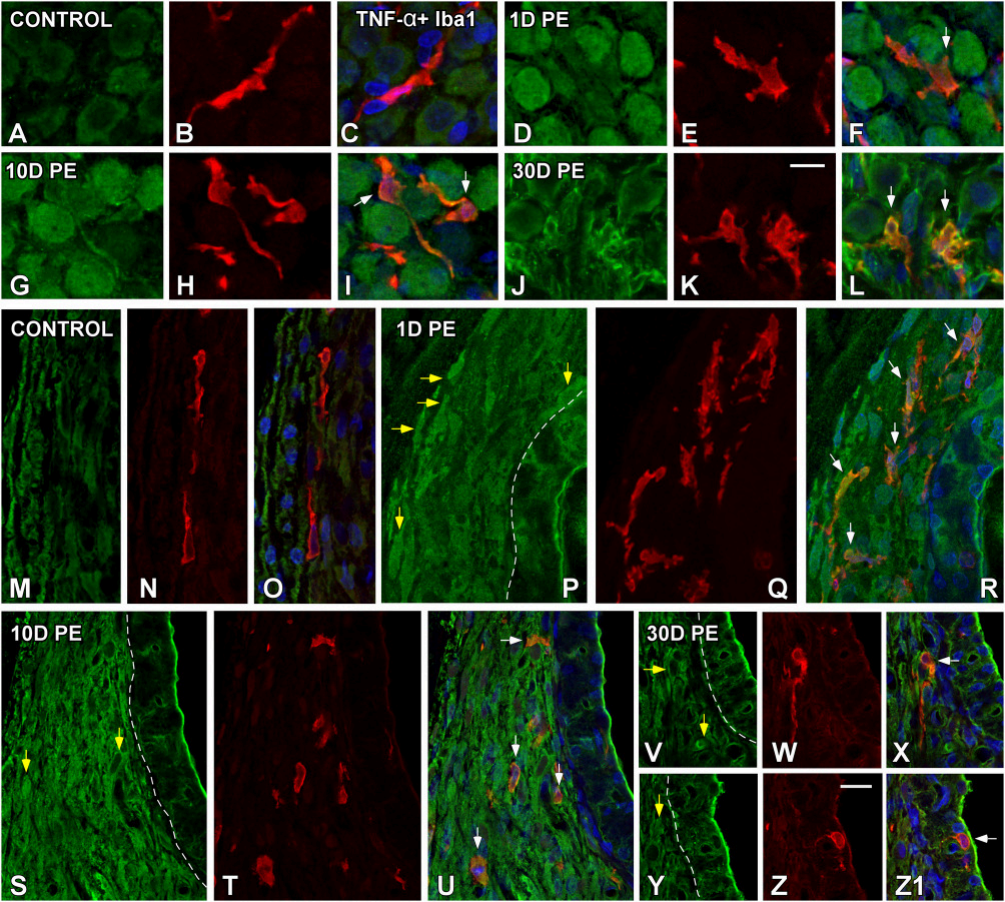
**Confocal images depicting the colocalization between TNF-α (green) and the microglial marker, Iba1 (red), in the SG (A–L), and SL (M–Z1) in control (***n*** = 5) and noise-exposed (***n*** = 5 for each group) rats**. In the SG, TNF-α producing cells in response to noise-exposure were neurons **(D,G,J)** and MLC **(E,H,K)** while in the SL, TNF-α protein was synthesized by MLC **(Q,T,W,Z)** and fibrocytes (yellow arrows in **P,S,V,Y**). White arrows point to double-stained cells in the SG and SL. Scale bars: 10 μm in **(K,Z)**.

**Figure 7:**
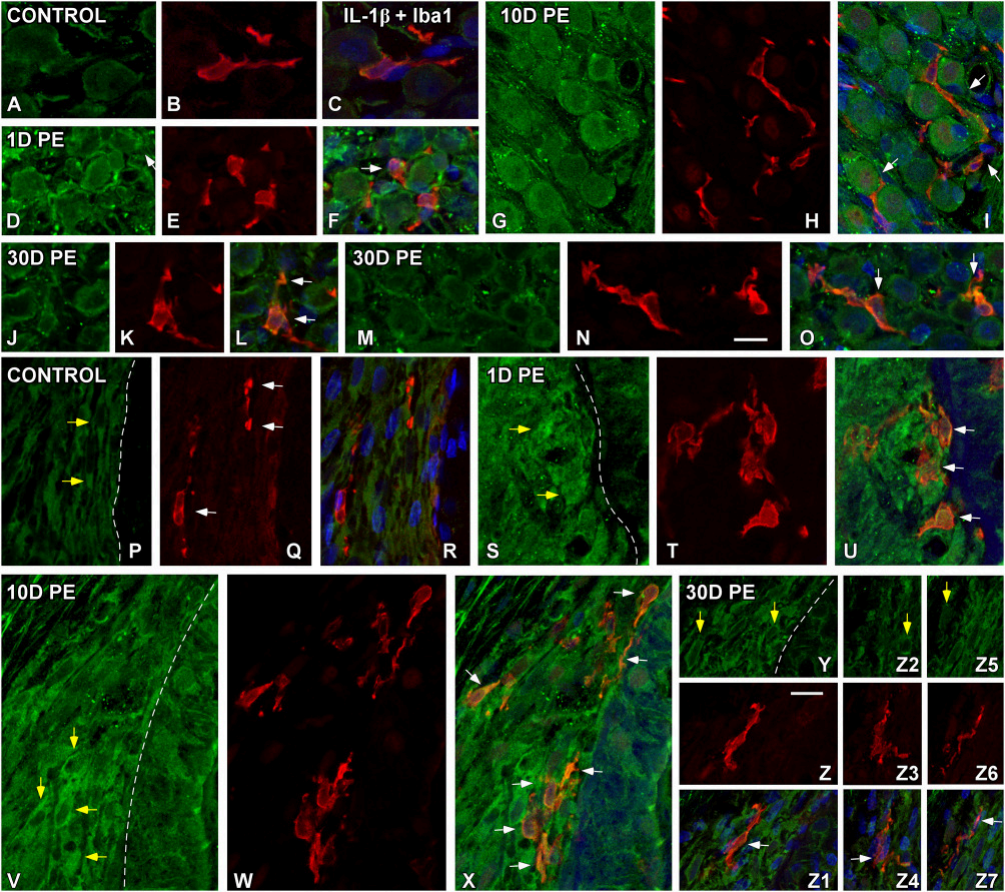
**Confocal images depicting the colocalization between IL-1β (green) and Iba1 (red) in the SG (A–O) and SL (P–Z7) in control (***n*** = 5) and noise-exposed (***n*** = 5 for each group) rats**. In the SG, IL-1β producing cells in response to noise-exposure were neurons **(D,G,J,M)** and MLC **(E,H,K,N)** while in the SL, IL-1β producing cell types were MLC **(T,W,Z,Z3,Z6)** and fibrocytes (yellow arrows in **S,V,Y,Z2,Z5**). White arrows point to double-stained cells in the SG and SL. Scale bars: 10 μm in **(N,Z)**.

At the protein level, significant increases in TNF-α (Figure 6) and IL-1β (Figure 7) staining were predominantly observed in the SG and the SL from noise-exposed rats when compared to unexposed rats. Immunohistochemically analysis of these tissues on day 1 and 10 post-exposure revealed an increased synthesis of these proinflammatory cytokines by neurons (green in Figures 6, 7) and activated MLC (red in Figures 6, 7) in the SG (arrows in Figures 6D–I, 7D–I). Although these increases in the SG were still present by 30D post-lesion, they were evident mostly in MLC and barely detectable in neurons (Figures 6J–L, 7J–O). In the noise-exposed SL, the sources of these cytokines were fibrocytes (yellow arrows in Figures 6, 7) and activated MLC (Figures 6Q,T,W,Z, 7T,W,Z,Z3,Z6). Although the majority of reactive MLC produced TNF-α or IL-1β in the SG and in the SL (white arrows in Figures 6, 7), many TNF-α and IL-1β-expressing fibrocytes did not colocalize with Iba1 (yellow arrows in Figures 6, 7). Note that cytokine levels in the control condition were very low in both the SG (Figures 6A–C, 7A–C) and SL (Figures 6M–O, 7P–R).

### Effects of noise exposure on microglia in the AVCN

**Figure 8:**
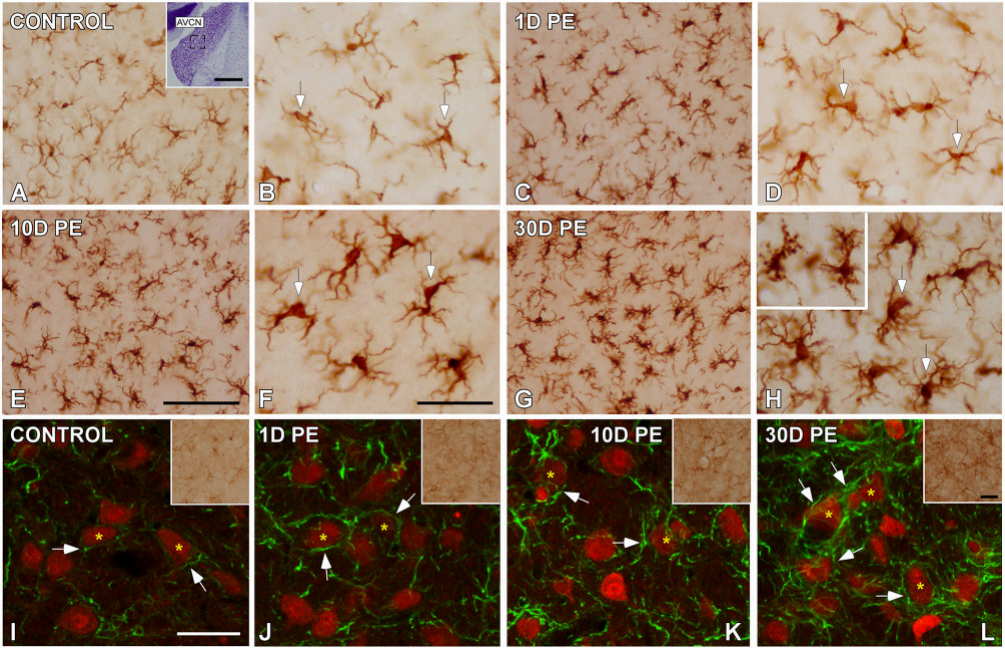
**Digital images showing the time course of glial activation in the AVCN in control (***n*** = 5) and noise-exposed (***n*** = 5 for each group) rats**. Microglial **(A–H)** and astroglial **(I–L)** activation responses increased progressively over time reaching maximum levels on day 30 post-exposure **(H,L)**. Microglia are indicated by arrows in **(A–H)** while appositions between cochlear nucleus neurons (red) and astrocytes (green) are designated by arrows in **(I–L)**. The inset in **(A)** shows the location of the AVCN, and the square box indicates the approximate locations of the fields represented in **(A–L)**. Scale bars: 50 μm in (**A;** inset); 100 μm in **(E)**; 50 μm in (**F,L;** inset); and 40 μm in **(I)**.

**Figure 9:**
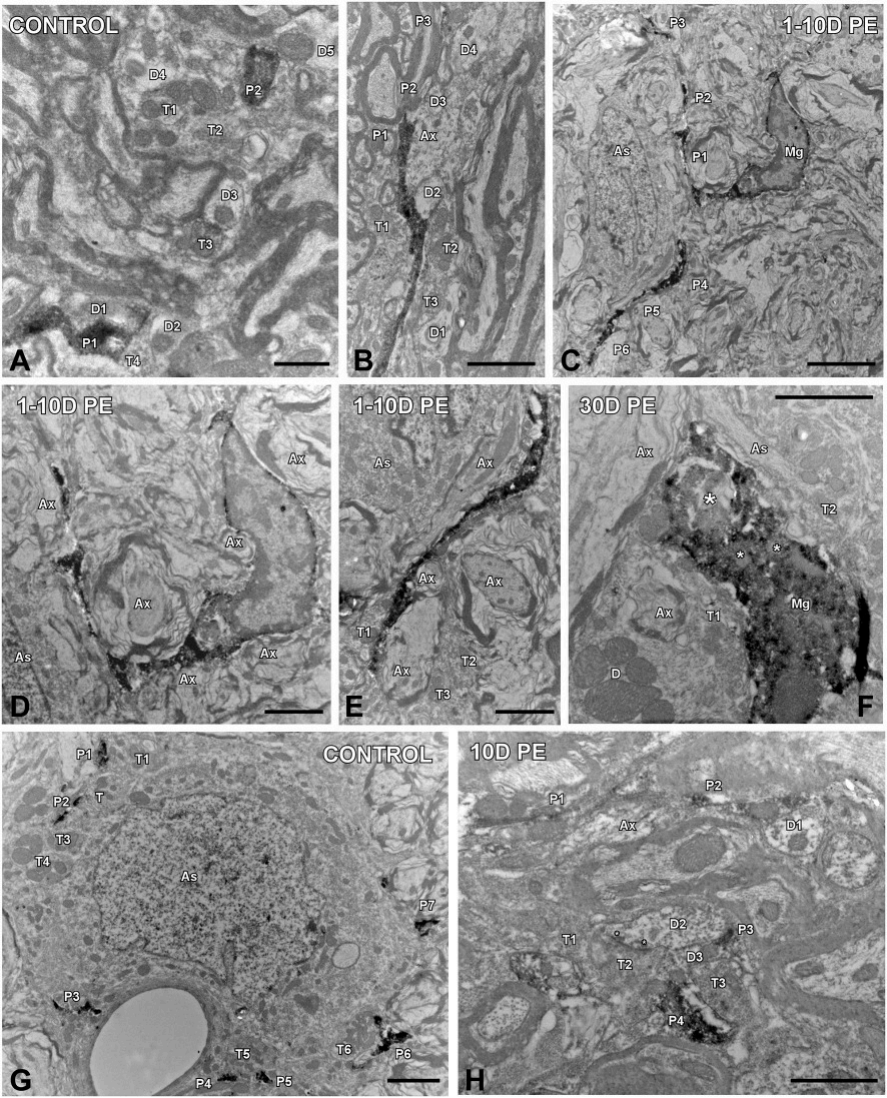
**Ultrastructure of microglia (A–F) and astrocytes (G,H) in the AVCN in control (***n*** = 4) and noise-exposed (***n*** = 4 for each group) rats**. In the control condition, microglial processes spread through the neuropil contacting multiple cellular elements **(A)**. Between 1 and 10D post-exposure **(C–E)**, dynamic microglia quickly associated with nearby elements preferentially axons and terminals in the vicinity. On day 30, a few microglial cells transformed into a phagocytic phenotype in which inclusion bodies (small asterisks) and engulfed material (large asterisk) were observed **(F)**. Note that this microglial cell was in close association with terminals, axons, dendrites and astrocytic processes. When compared to the control condition **(G)**, reactive astrocytic processes (P1–P4) on day 10 juxtaposed synapses (asterisks in **H**) and axons and terminals in the adjacent neuropil. As, astrocyte; Ax, Axon; D, D1-D5, dendrites; Mg, microglia, P1–P6, microglial/astroglial processes; T1–T6, terminals. Scale bars: 1 μm in **(A,H)**; 2 μm in **(B,D–G)**; 5 μm in **(C)**.

To determine how central auditory neurons respond to cochlear inflammation and subsequent sensorineural hearing loss, the contribution of microglia to maintain functional integrity, and prevent further damage was investigated in the CN. In unexposed rats, microglial cells were faintly stained and had multipolar and bipolar morphologies with long dynamic processes, which were indicative of the resting form (Figures 8A,B), as previously described (Fuentes-Santamaría et al., 2012, 2014). High-noise exposure led to progressive microglial activation and proliferation in the AVCN (Figure 8). Between 1D and 10D post-exposure, microglial cells turned into an alerted state characterized by larger cell bodies and cytoplasmic processes that were shorter and thicker (Figures 8C–F) when compared to unexposed rats (Figures 8A,B). On day 30, there was a heterogeneous population of microglia in which some of them had a bushy-like morphology characterized by enlarged cell bodies and thickened processes (Figure 8G and arrows in Figure 8H) which were suggestive of increased activation state when compared to earlier time points (Figures 8C–F) and unexposed (Figures 8A,B) rats. Upregulation of Iba1-inmunostaining at all time points evaluated was corroborated by significant increases in the mean gray levels of Iba1 in comparison with unexposed rats (Supplementary Table 7). To provide additional details of microglial phenotype following noise-exposure, the ultrastructural modifications of these cells were also evaluated (Figure 9). In the unexposed condition, proximal and distal microglial processes were frequently seen contacting multiple neuropil-associated elements (Figures 9A,B). Between 1 and 10D post-lesion, microglia was preferentially contacting presumptive lesioned axons and terminals (Figures 9C–E). On day 30, a few microglial cells had a very reactive phenotype characterized by enlarged cell bodies with multiple inclusions (small asterisks in Figure 9F) and remnants of engulfed membrane fragments (large asterisk in Figure 9F) in the cytoplasm, presumably from degenerating terminals. Note cell-cell direct interactions between irregular appearance microglia (Mg in Figure 9F), auditory terminals (T1 and T2 in Figure 9F) and astrocytic (As in Figure 9F) processes, suggesting noise-induced microglial remodeling of compromised synaptic connections.

### Effects of noise exposure on astrocytes in the AVCN

Given the role of astrocytes in the generation of trophic signals that stimulate recovery of auditory function, their temporal expression pattern in the CN was also assessed in response to noise overstimulation. In unexposed rats, astrocytes had a highly ramified appearance, and were distributed through the nucleus contacting other structural elements of the neuropil (arrows and asterisks in Figures 8I, 9G). Following noise stimulation, along with an astrocytes proliferation response that was more persistent with longer time points after the exposure, there was an increased occurrence of appositions between glia and AVCN neurons (arrows and asterisks in Figures 8J–L, 9H). Upregulation of GFAP-immunostaining was confirmed by quantifying the mean gray levels in noise-exposed and unexposed (Supplementary Table 7) rats. Note that synaptic terminals showing signs of degeneration (T1–T3 in Figure 9H) are making synaptic contacts (asterisks) with a dendrite (D2 in Figure 9H). One of the astrocytic processes (P4 in Figure 9H) is in close apposition with one of these terminals (T2 in Figure 9H).

### Interactions among CN neurons and glia following noise-exposure

**Figure 10:**
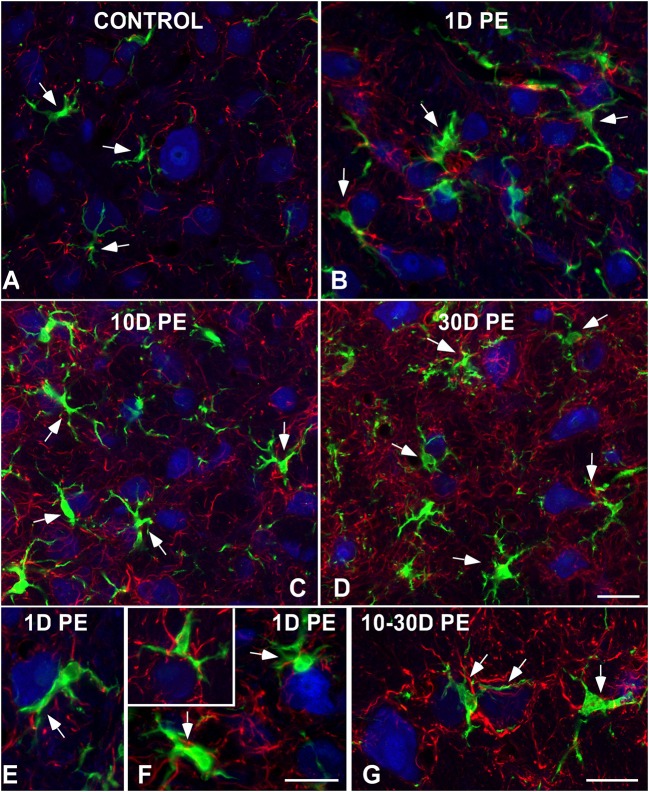
**Interactions between glia and neurons in the AVCN in control (A;***n*** = 5) and noise-exposed (***n*** = 5 for each group) rats (B–G)**. Microglial processes (green) and astrocytes (red) made multiple contacts simultaneously with cochlear nucleus neurons (blue) which increase in occurrence with longer time points post-exposure. Appositions between glial elements and neurons are indicated by arrows. Scale bars: 25 μm in **(D)**; 20 μm in **(F,G)**.

**Figure 11:**
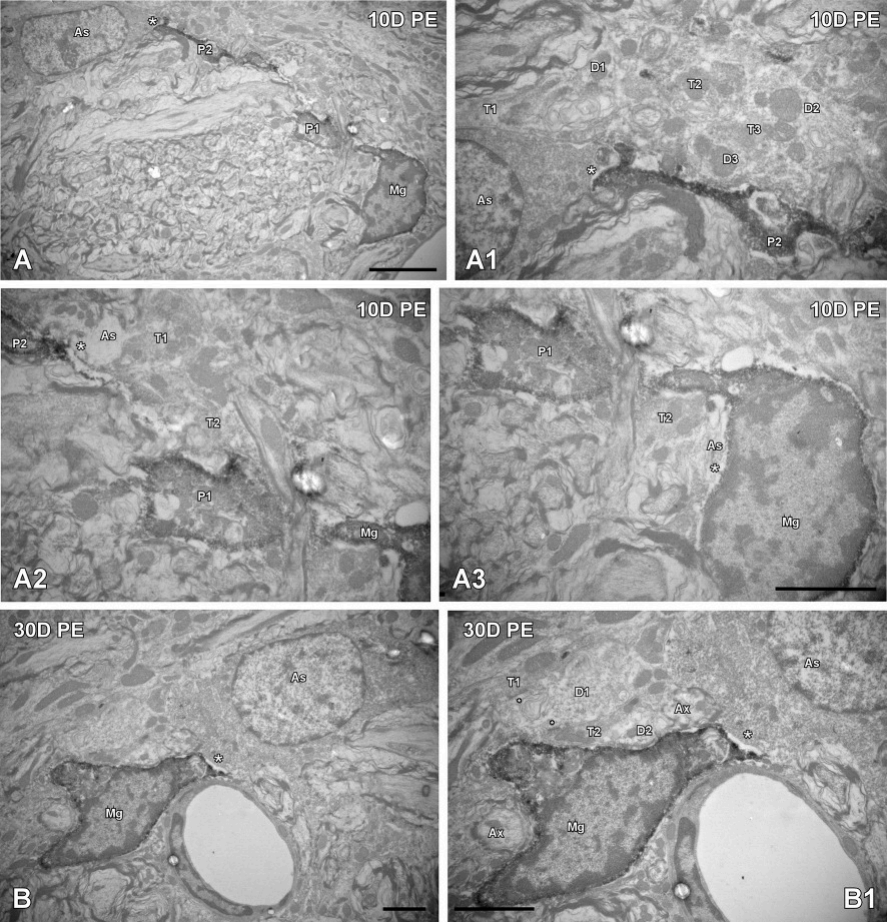
**Ultrastructural interactions among microglia, astrocytes and neurons in the AVCN are regulated by noise (***n*** = 4 for each group)**. In response to noise-exposure, reactive microglial processes came into close proximity with astrocytes, terminals, and synapse-associated elements at all time points evaluated **(A,B)**. Note that the perivascular microglial cell in **(B,B1)** is in close contact with an astrocyte (large asterisk) and is wrapping around a dendrite making synapses with multiple terminals (small asterisks). Although interactions among microglia, astrocytes and neuronal elements were observed at all time points post-exposure **(A–A3)**, they increased in frequency particularly on day 30 **(B,B1)**. Appositions between microglia and astrocytes are indicated by large asterisks while the small asterisks in **(B1)** point to synapses. Higher magnification images of **(A,B)** are represented in **(A1–A3, B1)**; respectively. As, astrocyte; Ax, axon; D1–D3, dendrites; Mg, microglia, P1–P2, microglial processes; T1–T3, terminals. Scale bars: 5 μm in **(A, B1)**; 2.5 μm in (**A3;** it also applies to **A1,A2**); 2 μm in **(B)**.

To further evaluate noise-induced alterations in neuronal-glial communication, the spatial relationship among microglia, neurons, and astrocytes was also examined by confocal and electron microscopy (Figures 10, 11). On day 1, microglial cells had shorter and thicker processes that came into close proximity with auditory neurons and astrocytes (arrows in Figures 10B,E,F) when compared to the unexposed condition (arrows in Figure 10A). By 10 and 30D post-exposure, there was a higher incidence of microglial contacts with other cellular elements (arrows in Figures 10C,D,G) including astrocytes (As, large asterisks in Figures 11A,B), axons (Ax, Figures 11A,B), synaptic terminals (T, Figures 11A,B), and dendrites (D, Figures 11A,B). Around the peak of glial maximum expression (day 30), the occurrence of glial-synapse contacts increased substantially (Figures 11B,B1). Note as irregular microglial appearance cell bodies were often juxtaposed with synapse-associated elements (pre- and post-synaptic elements and perisynaptic astrocyte). These glial direct interactions with synapses are suggestive of noise-induced microglial remodeling of synaptic connections. Note the close association between glial cells and blood capillaries (Figures 11B,B1). Quantitative analysis of these presumptive interactions revealed an increase in the colocalized area of both neurons and microglia and neurons and astrocytes at all time points post-exposure (Supplementary Table 8). This increase ranged from 30.84 to 92.73% for NeuN/Iba1 staining, and from 12.78 to 37.24% for NeuN/GFAP staining (Supplementary Table 8).

### TNF-α and IL-1β-expressing cells in the noise-exposed CN

**Figure 12:**
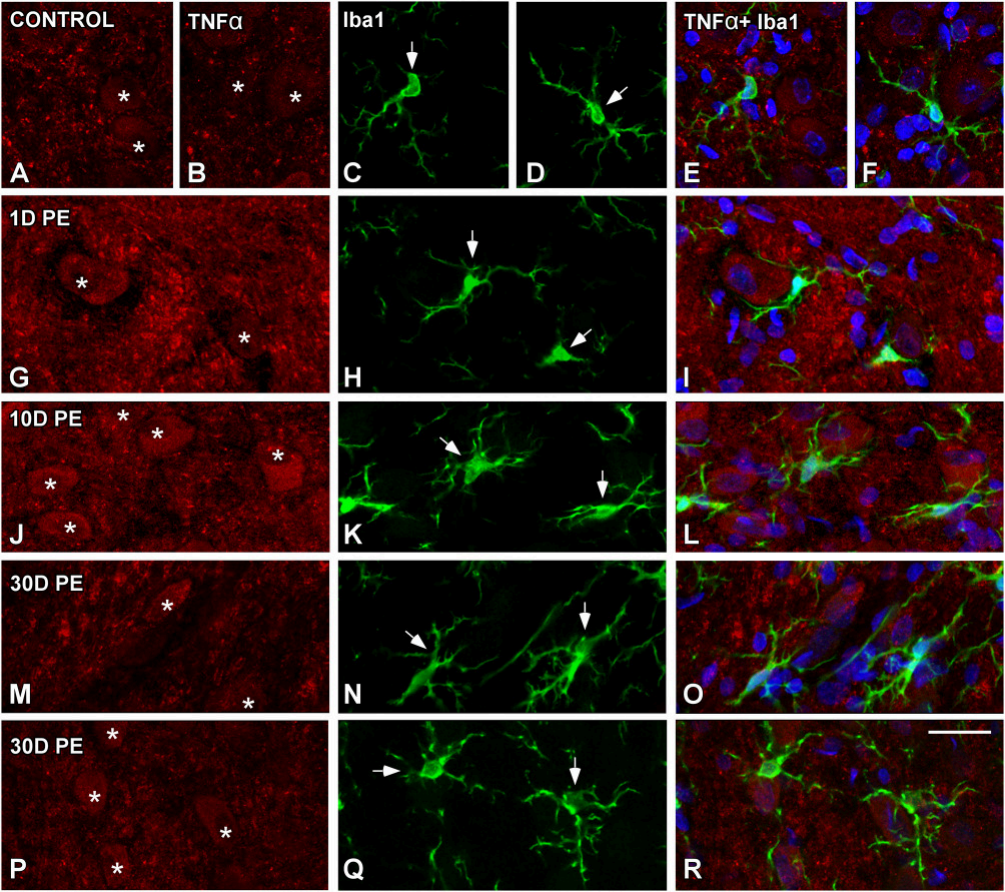
**Confocal images depicting the lack of colocalization between TNF-α and Iba1 in control (A–F) and noise-exposed (G–R) AVCN**. Note that TNF-α (red) was only expressed by auditory neurons (asterisks) and not by activated microglia (green) at all time points post-exposure (*n* = 5 for each group). Asterisks indicate TNF-α-containing neurons while arrows point to microglia. Scale bar: 25 μm in **(R)**.

**Figure 13:**
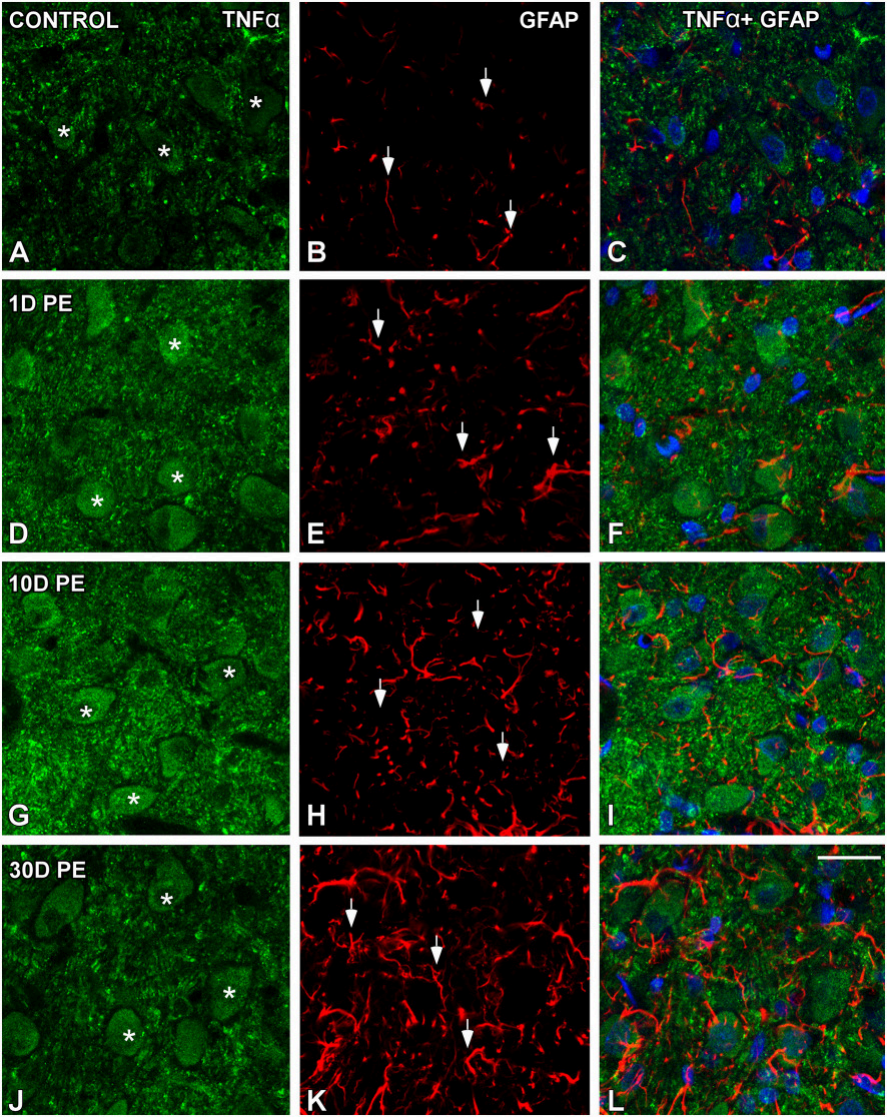
**Confocal images depicting the lack of colocalization between TNF-α and the astroglial marker, GFAP, in control (A–C) and noise-exposed (D–L) AVCN**. TNF-α (green) neither colocalized with GFAP (red) at any of the time points analyzed (*n* = 5 for each group). Asterisks indicate TNF-α-containing neurons while arrows point to astrocytes. Scale bar: 25 μm in **(L)**.

**Figure 14:**
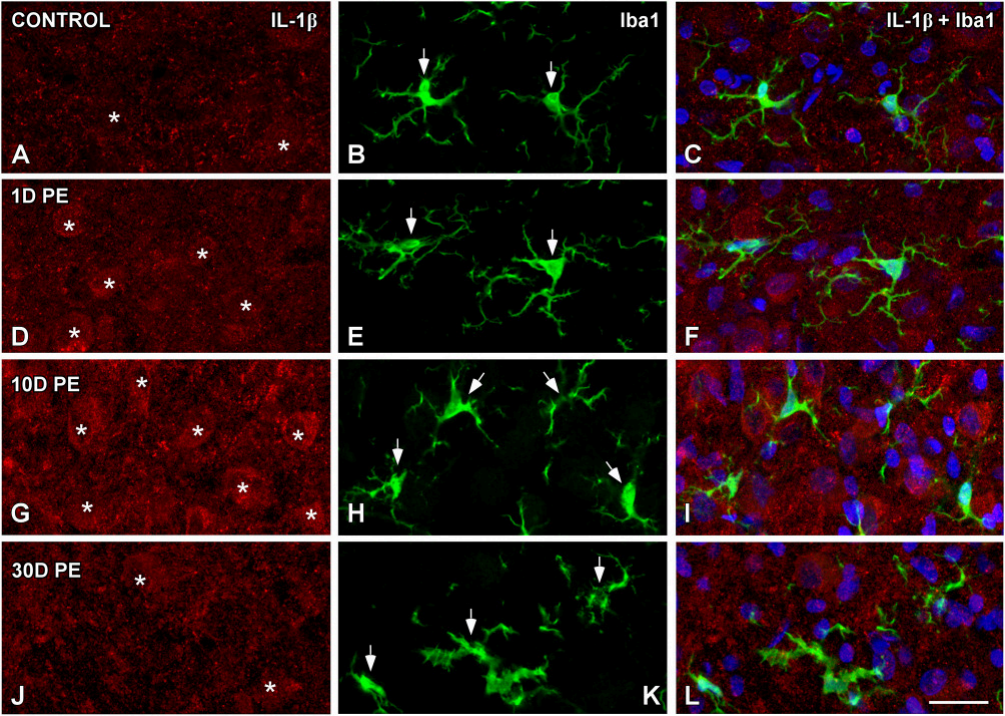
**Absence of colocalization between IL-1β and Iba1 in the AVCN in control rats (A–C) and following noise-exposure (D–L)**. Microglial (green) production of IL-1β (red) was not observed at any of the time points post-exposure (*n* = 5 for each group). Cochlear nucleus neurons were the sole source of this cytokine. Asterisks indicate TNF-α-containing neurons while arrows point to microglia. Scale bar: 25 μm in **(L)**.

**Figure 15:**
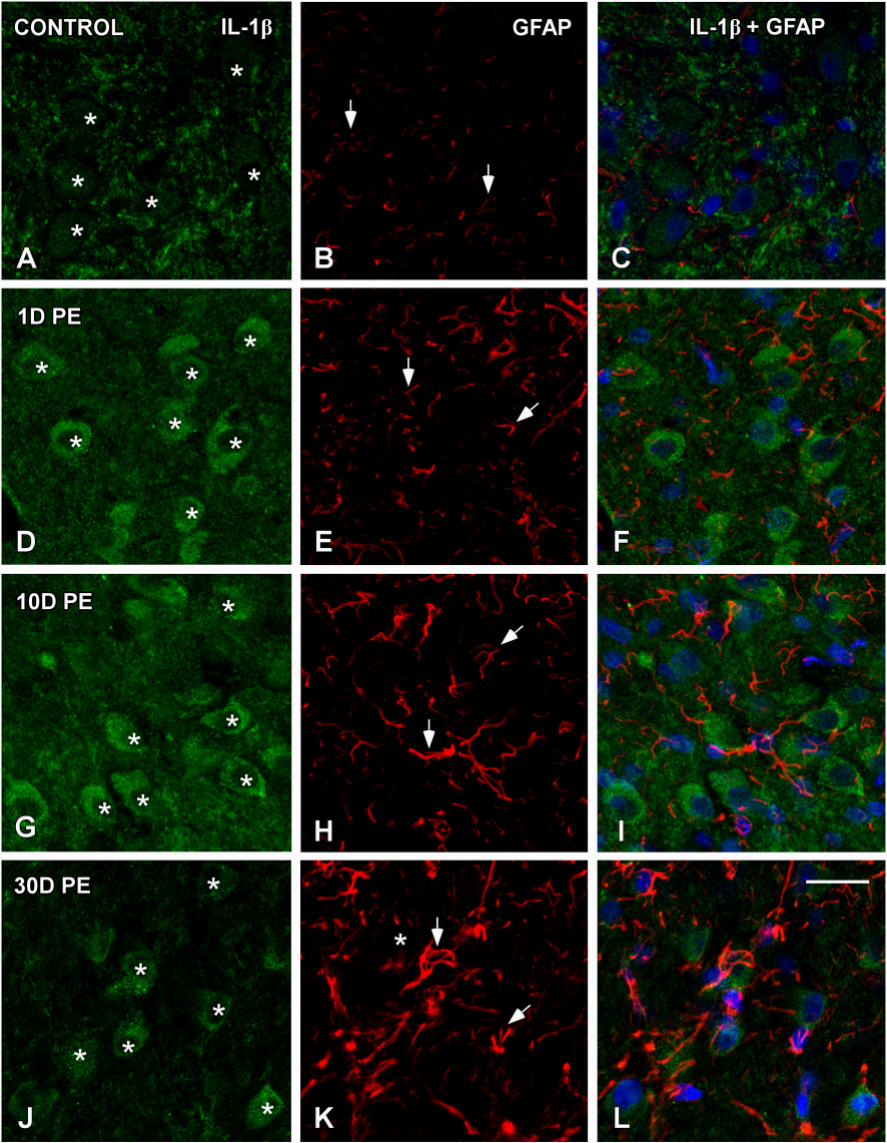
**Absence of colocalization between IL-1β and GFAP in the AVCN in control rats (A–C) and following noise-exposure (D–L)**. Astroglial (red) production of IL-1β (green) was not observed at any of the time points post-exposure (*n* = 5 for each group). IL-1β was primarily synthesized by cochlear nucleus neurons after the exposure. Asterisks indicate TNF-α-containing neurons while arrows point to astrocytes. Scale bar: 25 μm in **(L)**.

In the AVCN, TNF-α (Figures 12, 13), and IL-1β (Figures 14, 15) levels were increased within neurons at 1 (asterisks in Figures 12G, 13D, 14D, 15D) and 10D (asterisks in Figures 12J, 13G, 14G, 15G) post-lesion although they diminished progressively by day 30 (asterisks in Figures 12M,P, 13J, 14J, 15J) post-exposure. The absence of colocalization between TNF-α and microglia (arrows in Figure 12), TNF-α and astrocytes (arrows in Figure 13), IL-1β and microglia (arrows in Figure 14), and IL-1β and astrocytes (arrows in Figure 15) at any of the time points evaluated indicated that neurons were the only cell type overexpressing proinflammatory cytokines in the AVCN after noise-exposure.

## Discussion

Although the underlying mechanisms responsible for cochlear damage resulting from noise exposure have been extensively studied through the literature, much less attention has been given to the way that these noise-induced peripheral modifications translate into central changes. In this study, we have investigated the contribution of glial cells in regulating inflammation and resolution at peripheral and central levels in response to repetitive high-level noise exposure. Our results indicate that following such high intensity noise exposure there is a permanent hearing threshold shift of about 40 dB, which occurs concomitantly with OHC loss, prestin upregulation, and microglial activation in the cochlear structures most vulnerable to the lesion. Gene and protein expression levels of Iba1 in the SL and the SG were upregulated at 1 and 10D post-exposure and declined to basal levels at the longest time point (30D) assessed. A peripheral inflammatory response characterized by excessive synthesis of TNF-α, IL-1β, TGF-β, iNOS, and ICAM-1 was also observed on days 1 and 10 after noise exposure. Interestingly TIMP-1 expression peaked transiently at 1D post-lesion and quickly decreased between 10 and 30 days post-exposure. In the noise-exposed CN, microglial and astroglial responses were much more protracted reaching a maximum peak on day 30 which was associated with a significant increase in glial-neuronal interactions. These findings suggest that neurons, fibrocytes, and microglia represent the major cellular sources of TNF-α and IL-1β release in response to noise overexposure and highlight the fundamental role of these cytokines in the pathogenesis of noise-induced hearing loss.

Although the degree of damage on auditory function and structure depends on several factors such as noise properties (i.e., intensity, duration, and frequency), individual vulnerability to noise and survival time after the exposure, there is abundant evidence that noise overexposure leads to increases in auditory thresholds and morphological alterations in the inner ear (Syka and Popelár, 1980; Saunders et al., 1991; Bohne and Harding, 2000; Nordmann et al., 2000; Le Prell et al., 2003, 2007; Park et al., 2013; Chen et al., 2014; Sanz et al., 2015). While the initiating events associated with noise-induced pathology are thought to be direct mechanical damage, a metabolic stress associated with formation of excessive free radicals, or some combination of both, previous studies have demonstrated that noise-induced sensorineural hearing loss may depend on damage to a number of different cochlear cells, such as hair cells and their stereocilia, fibrocytes in the SL and the SLB, the SGN and the stria vascularis (Hu et al., 2000; Ou et al., 2000; Wang et al., 2002; Hirose and Liberman, 2003; Melgar-Rojas et al., 2015). In CBA/J mice, a single 30 min exposure to swept-sine noise (9–13 kHz at 120 dB) or 2 h exposure to broadband noise (2–20 kHz at 106 dB) result in loss of OHC in the basal and middle cochlear regions along with a permanent threshold shift (Chen et al., 2012; Sanz et al., 2015). Similarly, continuous noise exposure in rats (10 kHz at 100 dB) for 1 h during 10 consecutive days leads to ABR thresholds elevations, OHC loss in the intermediate and basal turns, stereocilia damage and alterations in the integrity of SGN (Fetoni et al., 2013, 2015). In the current study, adult Wistar rats were exposed to broadband noise (0.5–32 kHz, 118 dB SPL) for 4 h/day during 4 consecutive days to induce permanent auditory damage. Although this noise exposure paradigm is different from the one used in the aforementioned studies, as its uses a wider frequency range, its impact on auditory thresholds and cochlear cell types agrees with previous observations. Our findings also demonstrate that OHC loss and dysfunction occurs concomitantly with upregulation in prestin levels in the lateral wall of surviving noise-exposed hair cells at all time points post-exposure. Consistent with this view, elevated prestin protein and gene expression in residual OHC has been reported in noise-exposed rodents and proposed as a compensatory mechanism by which hearing loss can be partially recovered after insult (Chen, 2006; Mazurek et al., 2007; Xia et al., 2013; Parham, 2015).

The inner ear has its specialized resident immune system in which mainly phagocytic cells are involved in initiating and/or modulating inflammation in response to noise trauma (Abi-Hachem et al., 2010; Tan et al., 2013; Okano, 2014). During normal cochlear function, resident MLC serve fundamental roles as active sensors that detect minimal homeostatic disturbances (Lang et al., 2006; Okano et al., 2008; Sato et al., 2008). It has been reported previously that noise-induced damage to the cochlea triggers an inflammatory response that consists of early MLC activation and rapid synthesis and release of inflammatory mediators whose interplay facilitate migration of immune cells from the blood stream to the cochlea (Hirose et al., 2005; Fujioka et al., 2006; Tornabene et al., 2006; Miyao et al., 2008). In this regard, previous studies using CD45 as a leukocyte marker have demonstrated that the recruitment of circulating bone marrow-derived white blood cells takes place mainly in the SL which is considered a highly vulnerable structure to noise and a vascularized cellular system that may facilitate immune cells extravasation following noise-overstimulation (Hirose et al., 2005; Tornabene et al., 2006; Miyao et al., 2008). Our results demonstrate noise-induced MLC activation in the SL and also in the SG, the SLB and the cochlear nerve as early as 1D post-lesion, when microglia increase in number and exhibit a hypertrophic phenotype when compared to unexposed rats. These Iba1-stained cells were amoeboid or rounded in appearance resembling mature macrophages and were in close association with presumptive injured cochlear cells. This activation state remained elevated on day 10 and declined on day 30 to reach a near resting level. Although it is uncertain whether early activated phagocytes represent an activation and proliferation of a population of immune cells normally residing in the cochlea or an invasion of immune cells from the vasculature in response to injury, there is evidence of leucocytes infiltration to the SL between 2 and 4 days following noise exposure but not to the SG (Fujioka et al., 2006; Tornabene et al., 2006). Thus, at least for the SG, the observed cochlear phagocytes by 1D post-exposure were likely tissue resident cells. Our results agree with previous studies in which the authors by using additional monocyte/macrophage markers such us F4/80, CD68, and CX3CR1 reported a crucial role for cochlear MLC in regulating compromised auditory function (Hirose et al., 2005; Tornabene et al., 2006). However, in the current study we have observed a much higher degree of MLC activation than previously reported, particularly in the SL and the SG which correlates with a more extensive noise-induced tissue damage.

MLC, in addition to demonstrating a noise-induced cytoskeletal reorganization interact with other cell types by releasing inflammatory mediators (Hanisch, 2002; Kothur et al., 2016). Supporting this is the quantitative RT-PCR studies indicating noise-induced increases in the expression of proinflammatory cytokines (TNF-α, IL1β, and IL 6), chemokines (MCP-1, MCP5, and MIP), and the adhesion molecule ICAM-1 following a 2 h exposure at 124 dB (Fujioka et al., 2006; Tornabene et al., 2006; Tan et al., 2016). Besides cochlear macrophages, fibrocytes in the SL are essential participants in cochlear inflammation as they are primarily damaged and/or loss in a variety of animal models of noise-induced hearing loss and synthesize and release inflammation-related intercellular molecules (Wang et al., 2002; Murillo-Cuesta et al., 2015; Sanz et al., 2015). In this regard, *in vitro* investigations using cultured SL fibrocytes, have demonstrated that upon stimulation with proinflammatory cytokines, fibrocytes are able to secrete inflammatory mediators including IL1β, TNF-α, iNOS, MCP-1, MCP-2, ICAM-1, and VEGF (Yoshida et al., 1999; Ichimiya et al., 2000; Moriyama et al., 2007). Our results confirm the data observed in the aforementioned studies demonstrating noise-induced elevations in the expression levels of TNF-α, IL-1β, and ICAM-1 in the cochlea between 1 and 10D post-exposure and decreases on day 30, supporting a primary involvement of cytotoxic molecules and vascular adhesion molecules in mediating the acute phase of noise-induced cochlear inflammation. Evidence suggests that ICAM-1 expression, predominantly in the SL, is vital for the extravasation of immunocompetent cells into the damaged cochlea (Tornabene et al., 2006; Kel et al., 2013).

iNOS is a stress-induced neurotoxic molecule associated with a variety of pathophysiological disorders (Brown, 2007; Charriaut-Marlangue et al., 2013). Excessive levels of this mediator are linked to inflammatory processes which involve cytokine overproduction and infiltration of immune cells (Moncada and Bolaños, 2006; Pannu and Singh, 2006). Supporting this view, our observations reveal elevated levels of iNOS mRNA on days 1 and 10 following noise-exposure that progressively declined, reaching basal levels by day 30. Noise also induced a parallel increase in TGF-β cochlear gene expression. These early TGF-β elevations have been linked to regulation of the inflammatory response as this cytokine inhibits the production of proinflammatory cytokines and chemokines and induces adhesion molecules and matrix metalloproteinases that contribute to leukocytes activation (Li et al., 2006; Worthington et al., 2012). Moreover, the blockade of this anti-inflammatory cytokine by using specific inhibitors (P17 and P144) before or immediately after noise damage significantly ameliorates the inflammatory state and protects the cochlea from noise-induced hearing loss (Murillo-Cuesta et al., 2015). Concomitantly with the observed noise-induced upregulation in TNF-α, IL-1β, TGF-β, iNOS, and ICAM-1 mRNA expression, our results demonstrate a rapid and transient increase in TIMP-1 levels on day 1 and a remarkable decline with time. This increased expression is consistent with previous data reporting upregulation of TIM-1 gene expression at 2 h and 1 day after noise-exposure. At the protein level, TIMP-1 immunostaining was located in sensory cells with advanced nuclear condensation indicating that this endogenous matrix metalloproteinases inhibitor may modulate apoptosis in response to acoustic trauma (Hu et al., 2012).

Along with a noise-induced peripheral inflammatory response, we have reported a long-lasting response of reactive microglia and astrocytes in the AVCN that reached maximum levels on day 30 post-exposure. These results are agreement with a previous study in the CN demonstrating microglial activation in response to acoustic trauma (Baizer et al., 2015). In this latter study, the authors demonstrated that when rats were exposed to narrowband noise (bandwidth 100 Hz, 12 kHz, 126 SPL) for 2 h, OX6-immnostained cells distributed in both the dorsal and ventral subdivisions of the CN at 30 and 60 days and 6 months after the exposure. Such reactive cells have been proposed to act as scavengers, removing axonal debris.

The involvement of glial-related mechanisms in stabilizing the activity of CN networks following cochlear damage has been investigated by others (Lurie and Rubel, 1994; de Waele et al., 1996; Campos-Torres et al., 1999; Fuentes-Santamaría et al., 2012, 2013, 2014; Fredrich et al., 2013; Janz and Illing, 2014). In this regard, the CN is a highly plastic structure whose neurons, in response to a permanent threshold shift, adapt easily to the changing conditions by modifying their cellular properties. Elevations in spontaneous firing rates in VCN neurons have been observed immediately after noise trauma (Gröschel et al., 2014). This increased neuronal excitability may reflect an increase in cochlear peripheral activity that induces elevations in glutamate release at synapses between the SG and the CN (Gröschel et al., 2014). In support of this hypothesis, it has been suggested that glial activation regulates neuronal activity and synaptic strength by limiting excessive glutamate release and/or uptake (Patel et al., 2003). It is important to note that cochlear damage along with the increased neuronal activity in subcortical structures, such as the CN, have been causally associated with the auditory phantom sensation (tinnitus) that frequently occurs following intense noise exposure (Zacharek et al., 2002; Eggermont and Roberts, 2004; Lobarinas et al., 2008; Zheng et al., 2012; Rüttiger et al., 2013; Chen et al., 2014). Thus, considering that glial-related mechanisms are involved in the stabilization of CN activity after noise-induced cochlear damage, it might be expected that they would contribute to the pathogenesis, maintenance and/or regulation of noise-induced tinnitus. Consistent with these observations, our data demonstrate an increased number of interactions among CN neurons, microglia and astrocytes at all time points after the exposure. However, more studies will be needed to explore whether glial cells could represent a possible pharmacological target to treat this auditory perceptual disorder (Zacharek et al., 2002; Zheng et al., 2012).

One of the most noteworthy findings of this work is the fact that, TNF-α and IL-1β-producing cells in the SL and the SG were microglia, fibrocytes, and neurons, as opposed to the AVCN where solely neurons and not glial cells seemed to be responsible for the abnormal upregulation of TNF-α and IL-1β after noise trauma. Compelling evidence suggests that endangered neurons in the CN respond to modifications and/or deprivation of afferent activity by delivering signals that lead to phenotypic remodeling of nearby microglial and astrocytes which rapidly act to reduce tissue damage (Fuentes-Santamaría et al., 2012, 2013). In this regard, neuron-to-glia signaling is fundamental for glial activation, cytokines overproduction and neuronal excitability following irreversible cochlear damage. These observations together with our confocal and electron microscopy data indicate a reciprocal interaction among neurons, microglia and astrocytes that may facility activity-dependent release of secreted factors to maintain system homeostasis. TNF-α and IL-1β are multifunctional cytokines that, in addition to their essential role in the normal development of auditory function, regulate synaptic plasticity in response to microglia-mediated inflammation (Guo et al., 2007; Riazi et al., 2015). Thus, the fact that neurons themselves upregulate TNF-α and IL-1β levels in response to noise-exposure may represent an activity-dependent central mechanism to regulate glutamatergic transmission and maintain a stable level of excitability in the CN.

## Conclusions

In summary, our results together with previous observations suggest a complex interplay among different cytokine-producing cells in the noise-exposed auditory system (i.e., neurons, glia, fibrocytes, and infiltrating macrophages). Collectively, this plethora of different cell types orchestrate a sequence of rapidly occurring cellular events that modulate the initiation/progression of cochlear inflammation in the pathogenesis of noise-induced hearing loss. Also, our data suggest a primary role of TNF-α and IL1β signaling in the noise-exposed peripheral and central auditory system.

## Author contributions

All authors had full access to all the data in the study and take responsibility for the integrity of the data and the accuracy of the data analysis. Study concept and design: VF and JA. Acquisition of data: VF, JA, PM, and MG. Statistical analysis and interpretation of data: VF and JA. Drafting of the manuscript: VF and JA. Critical revision of the manuscript for important intellectual content: JA, VF, JM, and JJ.

## Funding

This study was supported by Programa I3 del Ministerio de Ciencia e Innovación (I320101590 to VF and I320101589 to JA), PROHEARING project of the 7th Framework Programme (FP7-HEALTH-2012-INNOVATON 304925 to JJ).

### Conflict of interest statement

The authors declare that the research was conducted in the absence of any commercial or financial relationships that could be construed as a potential conflict of interest.

## References

[B1] Abi-Hachem R. N. Zine A. Van De Water T. R. (2010). The injured cochlea as a target for inflammatory processes, initiation of cell death pathways and application of related otoprotectives strategies. Recent Patents CNS Drug Discov. 5, 147–163. 10.2174/157488910791213121 20167005

[B2] Alvarado J. C. Fuentes-Santamaría V. Gabaldón-Ull M. C. Blanco J. L. Juiz J. M. (2014). Wistar rats: a forgotten model of age-related hearing loss. Front. Aging Neurosci. 6:29. 10.3389/fnagi.2014.00029 24634657 PMC3942650

[B3] Alvarado J. C. Fuentes-Santamaría V. Gabaldón-Ull M. C. Jareño-Flores T. Miller J. M. Juiz J. M. (2016). Noise-induced “toughening” effect in wistar rats: enhanced auditory brainstem responses are related to calretinin and nitric oxide synthase upregulation. Front. Neuroanat. 10:19. 10.3389/fnana.2016.00019 27065815 PMC4815363

[B4] Alvarado J. C. Fuentes-Santamaría V. Henkel C. K. (2009a). Rapid modifications in calretinin immunostaining in the deep layers of the superior colliculus after unilateral cochlear ablation. Hear. Res. 247, 78–86. 10.1016/j.heares.2008.10.005 19017539

[B5] Alvarado J. C. Fuentes-Santamaría V. Henkel C. K. Brunso-Bechtold J. K. (2004). Alterations in calretinin immunostaining in the ferret superior olivary complex after cochlear ablation. J. Comp. Neurol. 470, 63–79. 10.1002/cne.11038 14755526

[B6] Alvarado J. C. Fuentes-Santamaría V. Jareño-Flores T. Blanco J. L. Juiz J. M. (2012). Normal variations in the morphology of auditory brainstem response (ABR) waveforms: a study in wistar rats. Neurosci. Res. 73, 302–311. 10.1016/j.neures.2012.05.001 22595234

[B7] Alvarado J. C. Stanford T. R. Rowland B. A. Vaughan J. W. Stein B. E. (2009b). Multisensory integration in the superior colliculus requires synergy among corticocollicular inputs. J. Neurosci. 29, 6580–6592. 10.1523/JNEUROSCI.0525-09.2009 19458228 PMC2805025

[B8] Alvarado J. C. Stanford T. R. Vaughan J. W. Stein B. E. (2007a). Cortex mediates multisensory but not unisensory integration in superior colliculus. J. Neurosci. 27, 12775–12786. 10.1523/JNEUROSCI.3524-07.2007 18032649 PMC6673293

[B9] Alvarado J. C. Vaughan J. W. Stanford T. R. Stein B. E. (2007b). Multisensory versus unisensory integration: contrasting modes in the superior colliculus. J. Neurophysiol. 97, 3193–3205. 10.1152/jn.00018.2007 17329632

[B10] Baizer J. S. Wong K. M. Manohar S. Hayes S. H. Ding D. Dingman R.. (2015). Effects of acoustic trauma on the auditory system of the rat: the role of microglia. Neuroscience 303, 299–311. 10.1016/j.neuroscience.2015.07.004 26162240 PMC4532607

[B11] Benson C. G. Gross J. S. Suneja S. K. Potashner S. J. (1997). Synaptophysin immunoreactivity in the cochlear nucleus after unilateral cochlear or ossicular removal. Synapse 25, 243–257. 10.1002/(SICI)1098-2396(199703)25:3<243::AID-SYN3>3.0.CO;2-B 9068122

[B12] Bhave S. A. Oesterle E. C. Coltrera M. D. (1998). Macrophage and microglia-like cells in the avian inner ear. J. Comp. Neurol. 398, 241–256. 10.1002/(SICI)1096-9861(19980824)398:2<241::AID-CNE6>3.0.CO;2-0 9700569

[B13] Bilak M. Kim J. Potashner S. J. Bohne B. A. Morest D. K. (1997). New growth of axons in the cochlear nucleus of adult chinchillas after acoustic trauma. Exp. Neurol. 147, 256–268. 10.1006/exnr.1997.6636 9344551

[B14] Bohne B. A. Harding G. W. (2000). Degeneration in the cochlea after noise damage: primary versus secondary events. Am. J. Otol. 21, 505–509. Available online at: http://journals.lww.com/otology-neurotology/Abstract/2000/07000/Degeneration_in_the_Cochlea_After_Noise_Damage_.9.aspx 10912695

[B15] Brown G. C. (2007). Mechanisms of inflammatory neurodegeneration: iNOS and NADPH oxidase. Biochem. Soc. Trans. 35, 1119–1121. 10.1042/BST0351119 17956292

[B16] Bruce-Keller A. J. (1999). Microglial-neuronal interactions in synaptic damage and recovery. J. Neurosci. Res. 58, 191–201. 10.1002/(SICI)1097-4547(19991001)58:1<191::AID-JNR17>3.0.CO;2-E 10491582

[B17] Bustin S. A. Benes V. Garson J. A. Hellemans J. Huggett J. Kubista M.. (2009). The MIQE guidelines: minimum information for publication of quantitative real-time PCR experiments. Clin. Chem. 55, 611–622. 10.1373/clinchem.2008.112797 19246619

[B18] Campos-Torres A. Vidal P. P. de Waele C. (1999). Evidence for a microglial reaction within the vestibular and cochlear nuclei following inner ear lesion in the rat. Neuroscience 92, 1475–1490. 10.1016/S0306-4522(99)00078-0 10426501

[B19] Cant N. B. Benson C. G. (2003). Parallel auditory pathways: projection patterns of the different neuronal populations in the dorsal and ventral cochlear nuclei. Brain Res. Bull. 60, 457–474. 10.1016/S0361-9230(03)00050-9 12787867

[B20] Cediel R. Riquelme R. Contreras J. Díaz A. Varela-Nieto I. (2006). Sensorineural hearing loss in insulin-like growth factor I-null mice: a new model of human deafness: hearing loss in Igf-1-mutant mice. Eur. J. Neurosci. 23, 587–590. 10.1111/j.1460-9568.2005.04584.x 16420467

[B21] Charriaut-Marlangue C. Bonnin P. Pham H. Loron G. Leger P.-L. Gressens P.. (2013). Nitric oxide signaling in the brain: a new target for inhaled nitric oxide? Ann. Neurol. 73, 442–448. 10.1002/ana.23842 23495069

[B22] Chen F. Q. Zheng H. W. Hill K. Sha S. H. (2012). Traumatic noise activates Rho-family GTPases through transient cellular energy depletion. J. Neurosci. Off. J. Soc. Neurosci. 32, 12421–12430. 10.1523/JNEUROSCI.6381-11.2012 22956833 PMC3445016

[B23] Chen G. D. (2006). Prestin gene expression in the rat cochlea following intense noise exposure. Hear. Res. 222, 54–61. 10.1016/j.heares.2006.08.011 17005342

[B24] Chen G. D. Decker B. Krishnan Muthaiah V. P. Sheppard A. Salvi R. (2014). Prolonged noise exposure-induced auditory threshold shifts in rats. Hear. Res. 317, 1–8. 10.1016/j.heares.2014.08.004 25219503 PMC4252814

[B25] Cullheim S. Thams S. (2007). The microglial networks of the brain and their role in neuronal network plasticity after lesion. Brain Res. Rev. 55, 89–96. 10.1016/j.brainresrev.2007.03.012 17509690

[B26] de Waele C. Campos Torres A. Josset P. Vidal P. P. (1996). Evidence for reactive astrocytes in rat vestibular and cochlear nuclei following unilateral inner ear lesion. Eur. J. Neurosci. 8, 2006–2018. 10.1111/j.1460-9568.1996.tb01344.x 8921291

[B27] Eggermont J. J. Roberts L. E. (2004). The neuroscience of tinnitus. Trends Neurosci. 27, 676–682. 10.1016/j.tins.2004.08.010 15474168

[B28] Fetoni A. R. De Bartolo P. Eramo S. L. M. Rolesi R. Paciello F. Bergamini C.. (2013). Noise-induced hearing loss (NIHL) as a target of oxidative stress-mediated damage: cochlear and cortical responses after an increase in antioxidant defense. J. Neurosci. 33, 4011–4023. 10.1523/JNEUROSCI.2282-12.2013 23447610 PMC6619303

[B29] Fetoni A. R. Troiani D. Petrosini L. Paludetti G. (2015). Cochlear injury and adaptive plasticity of the auditory cortex. Front. Aging Neurosci. 7:8. 10.3389/fnagi.2015.00008 25698966 PMC4318425

[B30] Fogal B. Hewett S. J. (2008). Interleukin-1beta: a bridge between inflammation and excitotoxicity? J. Neurochem. 106, 1–23. 10.1111/j.1471-4159.2008.05315.x 18315560

[B31] Fredrich M. Zeber A. C. Hildebrandt H. Illing R. B. (2013). Differential molecular profiles of astrocytes in degeneration and re-innervation after sensory deafferentation of the adult rat cochlear nucleus. Eur. J. Neurosci. 38, 2041–2056. 10.1111/ejn.12200 23581580

[B32] Fuentes-Santamaría V. Alvarado J. C. Brunso-Bechtold J. K. Henkel C. K. (2003). Upregulation of calretinin immunostaining in the ferret inferior colliculus after cochlear ablation. J. Comp. Neurol. 460, 585–596. 10.1002/cne.10676 12717716

[B33] Fuentes-Santamaría V. Alvarado J. C. Gabaldón-Ull M. C. Juiz J. M. (2013). Upregulation of insulin-like growth factor and interleukin 1β occurs in neurons but not in glial cells in the cochlear nucleus following cochlear ablation: upregulation of IGF-1 and IL-1β in Cochlear Nucleus. J. Comp. Neurol. 521, 3478–3499. 10.1002/cne.23362 23681983

[B34] Fuentes-Santamaría V. Alvarado J. C. Henkel C. K. Brunso-Bechtold J. K. (2007a). Cochlear ablation in adult ferrets results in changes in insulin-like growth factor-1 and synaptophysin immunostaining in the cochlear nucleus. Neuroscience 148, 1033–1047. 10.1016/j.neuroscience.2007.07.026 17764853

[B35] Fuentes-Santamaría V. Alvarado J. C. Herranz A. S. García-Atarés N. López D. E. (2007b). Morphologic and neurochemical alterations in the superior colliculus of the genetically epilepsy-prone hamster (GPG/Vall). Epilepsy Res. 75, 206–219. 10.1016/j.eplepsyres.2007.06.005 17628427

[B36] Fuentes-Santamaría V. Alvarado J. C. Juiz J. M. (2012). Long-term interaction between microglial cells and cochlear nucleus neurons after bilateral cochlear ablation. J. Comp. Neurol. 520, 2974–2990. 10.1002/cne.23088 22351306

[B37] Fuentes-Santamaría V. Alvarado J. C. López-Muñoz D. F. Melgar-Rojas P. Gabaldón-Ull M. C. Juiz J. M. (2014). Glia-related mechanisms in the anteroventral cochlear nucleus of the adult rat in response to unilateral conductive hearing loss. Front. Neurosci. 8:319. 10.3389/fnins.2014.00319 25352772 PMC4195288

[B38] Fuentes-Santamaria V. Alvarado J. C. Stein B. E. McHaffie J. G. (2008). Cortex contacts both output neurons and nitrergic interneurons in the superior colliculus: direct and indirect routes for multisensory integration. Cereb. Cortex. 18, 1640–1652. 10.1093/cercor/bhm192 18003596 PMC2853375

[B39] Fuentes-Santamaría V. Cantos R. Alvarado J. C. García-Atarés N. López D. E. (2005). Morphologic and neurochemical abnormalities in the auditory brainstem of the genetically epilepsy-prone hamster (GPG/Vall). Epilepsia 46, 1027–1045. 10.1111/j.1528-1167.2005.68104.x 16026555

[B40] Fujioka M. Kanzaki S. Okano H. J. Masuda M. Ogawa K. Okano H. (2006). Proinflammatory cytokines expression in noise-induced damaged cochlea. J. Neurosci. Res. 83, 575–583. 10.1002/jnr.20764 16429448

[B41] Garcia-Pino E. Caminos E. Juiz J. M. (2009). KCNQ5 reaches synaptic endings in the auditory brainstem at hearing onset and targeting maintenance is activity-dependent. J. Comp. Neurol. 518, 1301–1314. 10.1002/cne.22276 20151361

[B42] Gourévitch B. Doisy T. Avillac M. Edeline J.-M. (2009). Follow-up of latency and threshold shifts of auditory brainstem responses after single and interrupted acoustic trauma in guinea pig. Brain Res. 1304, 66–79. 10.1016/j.brainres.2009.09.041 19766602

[B43] Gröschel M. Ryll J. Götze R. Ernst A. Basta D. (2014). Acute and long-term effects of noise exposure on the neuronal spontaneous activity in cochlear nucleus and inferior colliculus brain slices. Biomed Res. Int. 2014:909260. 10.1155/2014/909260 25110707 PMC4119618

[B44] Guo W. Wang H. Watanabe M. Shimizu K. Zou S. LaGraize S. C.. (2007). Glial-cytokine-neuronal interactions underlying the mechanisms of persistent pain. J. Neurosci. Off. J. Soc. Neurosci. 27, 6006–6018. 10.1523/JNEUROSCI.0176-07.2007 17537972 PMC2676443

[B45] Hanisch U. K. (2002). Microglia as a source and target of cytokines. Glia 40, 140–155. 10.1002/glia.10161 12379902

[B46] Hanisch U. K. Kettenmann H. (2007). Microglia: active sensor and versatile effector cells in the normal and pathologic brain. Nat. Neurosci. 10, 1387–1394. 10.1038/nn1997 17965659

[B47] Hildebrandt H. Hoffmann N. A. Illing R. B. (2011). Synaptic reorganization in the adult rat's ventral cochlear nucleus following its total sensory deafferentation. PLoS ONE 6:e23686. 10.1371/journal.pone.0023686 21887295 PMC3161744

[B48] Hirose K. Discolo C. M. Keasler J. R. Ransohoff R. (2005). Mononuclear phagocytes migrate into the murine cochlea after acoustic trauma. J. Comp. Neurol. 489, 180–194. 10.1002/cne.20619 15983998

[B49] Hirose K. Liberman M. C. (2003). Lateral wall histopathology and endocochlear potential in the noise-damaged mouse cochlea. J. Assoc. Res. Otolaryngol. 4, 339–352. 10.1007/s10162-002-3036-4 14690052 PMC1805786

[B50] Hu B. H. Cai Q. Hu Z. Patel M. Bard J. Jamison J.. (2012). Metalloproteinases and their associated genes contribute to the functional integrity and noise-induced damage in the cochlear sensory epithelium. J. Neurosci. 32, 14927–14941. 10.1523/JNEUROSCI.1588-12.2012 23100416 PMC3521496

[B51] Hu B. H. Guo W. Wang P. Y. Henderson D. Jiang S. C. (2000). Intense noise-induced apoptosis in hair cells of guinea pig cochleae. Acta Otolaryngol. 120, 19–24. 10.1080/000164800760370774 10779180

[B52] Hu B. H. Henderson D. (1997). Changes in F-actin labeling in the outer hair cell and the Deiters cell in the chinchilla cochlea following noise exposure. Hear. Res. 110, 209–218. 10.1016/S0378-5955(97)00075-0 9282903

[B53] Ichimiya I. Yoshida K. Hirano T. Suzuki M. Mogi G. (2000). Significance of spiral ligament fibrocytes with cochlear inflammation. Int. J. Pediatr. Otorhinolaryngol. 56, 45–51. 10.1016/S0165-5876(00)00408-0 11074115

[B54] Ichimiya I. Yoshida K. Suzuki M. Mogi G. (2003). Expression of adhesion molecules by cultured spiral ligament fibrocytes stimulated with proinflammatory cytokines. Ann. Otol. Rhinol. Laryngol. 112, 722–728. 10.1177/000348940311200813 12940672

[B55] Janz P. Illing R.-B. (2014). A role for microglial cells in reshaping neuronal circuitry of the adult rat auditory brainstem after its sensory deafferentation. J. Neurosci. Res. 92, 432–445. 10.1002/jnr.23334 24446187

[B56] Kel G. E. Tan J. Eastwood H. T. Wongprasartsuk S. O'Leary S. J. (2013). Early cochlear response and ICAM-1 expression to cochlear implantation. Otol. Neurotol. Off. Publ. Am. Otol. Soc. Am. Neurotol. Soc. Eur. Acad. Otol. Neurotol. 34, 1595–1602. 10.1097/MAO.0b013e31828f4929 23928509

[B57] Kim J. H. Min K. J. Seol W. Jou I. Joe E. H. (2010). Astrocytes in injury states rapidly produce anti-inflammatory factors and attenuate microglial inflammatory responses. J. Neurochem. 115, 1161–1171. 10.1111/j.1471-4159.2010.07004.x 21039520

[B58] Kim J. J. Gross J. Potashner S. J. Morest D. K. (2004). Fine structure of long-term changes in the cochlear nucleus after acoustic overstimulation: chronic degeneration and new growth of synaptic endings. J. Neurosci. Res. 77, 817–828. 10.1002/jnr.20212 15334600

[B59] Kothur K. Wienholt L. Brilot F. Dale R. C. (2016). CSF cytokines/chemokines as biomarkers in neuroinflammatory CNS disorders: a systematic review. Cytokine 77, 227–237. 10.1016/j.cyto.2015.10.001 26463515

[B60] Kujawa S. G. Liberman M. C. (2015). Synaptopathy in the noise-exposed and aging cochlea: primary neural degeneration in acquired sensorineural hearing loss. Hear. Res. 330, 191–199. 10.1016/j.heares.2015.02.009 25769437 PMC4567542

[B61] Ladrech S. Wang J. Simonneau L. Puel J.-L. Lenoir M. (2007). Macrophage contribution to the response of the rat organ of Corti to amikacin. J. Neurosci. Res. 85, 1970–1979. 10.1002/jnr.21335 17497672

[B62] Lang H. Ebihara Y. Schmiedt R. A. Minamiguchi H. Zhou D. Smythe N.. (2006). Contribution of bone marrow hematopoietic stem cells to adult mouse inner ear: mesenchymal cells and fibrocytes. J. Comp. Neurol. 496, 187–201. 10.1002/cne.20929 16538683 PMC2561311

[B63] Le Prell C. G. Dolan D. F. Schacht J. Miller J. M. Lomax M. I. Altschuler R. A. (2003). Pathways for protection from noise induced hearing loss. Noise Health 5, 1–17. Available online at: http://www.noiseandhealth.org/article.asp?issn=1463-1741;year=2003;volume=5;issue=20;spage=1;epage=17;aulast=Le 14558888

[B64] Le Prell C. G. Yamashita D. Minami S. B. Yamasoba T. Miller J. M. (2007). Mechanisms of noise-induced hearing loss indicate multiple methods of prevention. Hear. Res. 226, 22–43. 10.1016/j.heares.2006.10.006 17141991 PMC1995566

[B65] Li M. O. Wan Y. Y. Sanjabi S. Robertson A.-K. L. Flavell R. A. (2006). Transforming growth factor-β regulation of immune responses. Annu. Rev. Immunol. 24, 99–146. 10.1146/annurev.immunol.24.021605.090737 16551245

[B66] Lobarinas E. Sun W. Stolzberg D. Lu J. Salvi R. (2008). Human brain imaging of tinnitus and animal models. Semin. Hear. 29, 333–349. 10.1055/s-0028-1095893 19122834 PMC2613289

[B67] Luo X.-G. Chen S.-D. (2012). The changing phenotype of microglia from homeostasis to disease. Transl. Neurodegener. 1:9. 10.1186/2047-9158-1-9 23210447 PMC3514090

[B68] Lurie D. I. Rubel E. W. (1994). Astrocyte proliferation in the chick auditory brainstem following cochlea removal. J. Comp. Neurol. 346, 276–288. 10.1002/cne.903460207 7962719

[B69] Madinier A. Bertrand N. Mossiat C. Prigent-Tessier A. Beley A. Marie C.. (2009). Microglial involvement in neuroplastic changes following focal brain ischemia in rats. PLoS ONE 4:e8101. 10.1371/journal.pone.0008101 19956568 PMC2779656

[B70] Mason J. L. Suzuki K. Chaplin D. D. Matsushima G. K. (2001). Interleukin-1beta promotes repair of the CNS. J. Neurosci. Off. J. Soc. Neurosci. 21, 7046–7052. Available online at: http://www.jneurosci.org/content/21/18/7046.long 10.1523/JNEUROSCI.21-18-07046.2001 PMC6762979 11549714

[B71] Mazurek B. Haupt H. Amarjargal N. Yarin Y. M. Machulik A. Gross J. (2007). Up-regulation of prestin mRNA expression in the organs of Corti of guinea pigs and rats following unilateral impulse noise exposure. Hear. Res. 231, 73–83. 10.1016/j.heares.2007.05.008 17592749

[B72] Melgar-Rojas P. Alvarado J. C. Fuentes–Santamaría V. Gabaldón–Ull M. C. Juiz J. M. (2015). Validation of reference genes for RT–qPCR analysis in noise–induced hearing loss: a study in Wistar rat. PLoS ONE 10:e0138027. 10.1371/journal.pone.0138027 26366995 PMC4569353

[B73] Meredith M. A. Stein B. E. (1983). Interactions among converging sensory inputs in the superior colliculus. Science 221, 389–391. 10.1126/science.6867718 6867718

[B74] Min K. J. Yang M. Kim S. U. Jou I. Joe E. (2006). Astrocytes induce hemeoxygenase-1 expression in microglia: a feasible mechanism for preventing excessive brain inflammation. J. Neurosci. Off. J. Soc. Neurosci. 26, 1880–1887. 10.1523/JNEUROSCI.3696-05.2006 16467537 PMC6793633

[B75] Minami S. B. Yamashita D. Ogawa K. Schacht J. Miller J. M. (2007). Creatine and tempol attenuate noise-induced hearing loss. Brain Res. 1148, 83–89. 10.1016/j.brainres.2007.02.021 17359945 PMC2680083

[B76] Miyao M. Firestein G. S. Keithley E. M. (2008). Acoustic trauma augments the cochlear immune response to antigen. Laryngoscope 118, 1801–1808. 10.1097/MLG.0b013e31817e2c27 18806477 PMC2832795

[B77] Moncada S. Bolaños J. P. (2006). Nitric oxide, cell bioenergetics and neurodegeneration. J. Neurochem. 97, 1676–1689. 10.1111/j.1471-4159.2006.03988.x 16805776

[B78] Morest D. K. Kim J. Potashner S. J. Bohne B. A. (1998). Long-term degeneration in the cochlear nerve and cochlear nucleus of the adult chinchilla following acoustic overstimulation. Microsc. Res. Tech. 41, 205–216. 9605338 10.1002/(SICI)1097-0029(19980501)41:3<205::AID-JEMT4>3.0.CO;2-S

[B79] Moriyama M. Yoshida K. Ichimiya I. Suzuki M. (2007). Nitric oxide production from cultured spiral ligament fibrocytes: effects of corticosteroids. Acta Otolaryngol. (Stockh.) 127, 676–681. 10.1080/00016480600987859 17573561

[B80] Mrak R. E. Griffin W. S. (2005). Glia and their cytokines in progression of neurodegeneration. Neurobiol. Aging 26, 349–354. 10.1016/j.neurobiolaging.2004.05.010 15639313

[B81] Mugnaini E. Warr W. B. Osen K. K. (1980). Distribution and light microscopic features of granule cells in the cochlear nuclei of cat, rat, and mouse. J. Comp. Neurol. 191, 581–606. 10.1002/cne.901910406 6158528

[B82] Muly S. (2002). Synaptophysin in the cochlear nucleus following acoustic trauma. Exp. Neurol. 177, 202–221. 10.1006/exnr.2002.7963 12429223

[B83] Muly S. M. Gross J. S. Potashner S. J. (2004). Noise trauma alters D-[3H]aspartate release and AMPA binding in chinchilla cochlear nucleus. J. Neurosci. Res. 75, 585–596. 10.1002/jnr.20011 14743442

[B84] Murillo-Cuesta S. Rodríguez-de la Rosa L. Contreras J. Celaya A. M. Camarero G. Rivera T.. (2015). Transforming growth factor Î^2^1 inhibition protects from noise-induced hearing loss. Front. Aging Neurosci. 7:32. 10.3389/fnagi.2015.00032 25852546 PMC4367183

[B85] Nordmann A. S. Bohne B. A. Harding G. W. (2000). Histopathological differences between temporary and permanent threshold shift. Hear. Res. 139, 13–30. 10.1016/S0378-5955(99)00163-X 10601709

[B86] Okano T. (2014). Immune system of the inner ear as a novel therapeutic target for sensorineural hearing loss. Front. Pharmacol. 5:205. 10.3389/fphar.2014.00205 25228882 PMC4151383

[B87] Okano T. Nakagawa T. Kita T. Kada S. Yoshimoto M. Nakahata T.. (2008). Bone marrow-derived cells expressing Iba1 are constitutively present as resident tissue macrophages in the mouse cochlea. J. Neurosci. Res. 86, 1758–1767. 10.1002/jnr.21625 18253944

[B88] Ou H. C. Bohne B. A. Harding G. W. (2000). Noise damage in the C57BL/CBA mouse cochlea. Hear. Res. 145, 111–122. 10.1016/S0378-5955(00)00081-2 10867283

[B89] Pannu R. Singh I. (2006). Pharmacological strategies for the regulation of inducible nitric oxide synthase: neurodegenerative versus neuroprotective mechanisms. Neurochem. Int. 49, 170–182. 10.1016/j.neuint.2006.04.010 16765486

[B90] Parham K. (2015). Prestin as a biochemical marker for early detection of acquired sensorineural hearing loss. Med. Hypotheses 85, 130–133. 10.1016/j.mehy.2015.04.015 25920562

[B91] Park S. N. Back S. A. Park K. H. Seo J. H. Noh H. I. Akil O.. (2013). Comparison of functional and morphologic characteristics of mice models of noise-induced hearing loss. Auris Nasus Larynx 40, 11–17. 10.1016/j.anl.2011.11.008 22364846

[B92] Patel A. B. De Graaf R. A. Mason G. F. Rothman D. L. Shulman R. G. Behar K. L. (2003). Coupling of glutamatergic neurotransmission and neuronal glucose oxidation over the entire range of cerebral cortex activity. Ann. N.Y. Acad. Sci. 1003, 452–453. 10.1196/annals.1300.050 14684486

[B93] Rasmussen S. Wang Y. Kivisäkk P. Bronson R. T. Meyer M. Imitola J.. (2007). Persistent activation of microglia is associated with neuronal dysfunction of callosal projecting pathways and multiple sclerosis-like lesions in relapsing–remitting experimental autoimmune encephalomyelitis. Brain J. Neurol. 130, 2816–2829. 10.1093/brain/awm219 17890734

[B94] Riazi K. Galic M. A. Kentner A. C. Reid A. Y. Sharkey K. A. Pittman Q. J. (2015). Microglia-dependent alteration of glutamatergic synaptic transmission and plasticity in the hippocampus during peripheral inflammation. J. Neurosci. Off. J. Soc. Neurosci. 35, 4942–4952. 10.1523/JNEUROSCI.4485-14.2015 25810524 PMC6705378

[B95] Rüttiger L. Singer W. Panford-Walsh R. Matsumoto M. Lee S. C. Zuccotti A.. (2013). The reduced cochlear output and the failure to adapt the central auditory response causes tinnitus in noise exposed rats. PLoS ONE 8:e57247. 10.1371/journal.pone.0057247 23516401 PMC3596376

[B96] Sanz L. Murillo-Cuesta S. Cobo P. Cediel-Algovia R. Contreras J. Rivera T.. (2015). Swept-sine noise-induced damage as a hearing loss model for preclinical assays. Front. Aging Neurosci. 7:7. 10.3389/fnagi.2015.00007 25762930 PMC4329813

[B97] Sato E. Shick H. E. Ransohoff R. M. Hirose K. (2008). Repopulation of cochlear macrophages in murine hematopoietic progenitor cell chimeras: the role of CX3CR1. J. Comp. Neurol. 506, 930–942. 10.1002/cne.21583 18085589

[B98] Saunders J. C. Cohen Y. E. Szymko Y. M. (1991). The structural and functional consequences of acoustic injury in the cochlea and peripheral auditory system: a five year update. J. Acoust. Soc. Am. 90, 136–146. 10.1121/1.401307 1880281

[B99] Schmittgen T. D. Livak K. J. (2008). Analyzing real-time PCR data by the comparative C(T) method. Nat. Protoc. 3, 1101–1108. 10.1038/nprot.2008.73 18546601

[B100] Shih A. Y. Fernandes H. B. Choi F. Y. Kozoriz M. G. Liu Y. Li P.. (2006). Policing the police: astrocytes modulate microglial activation. J. Neurosci. Off. J. Soc. Neurosci. 26, 3887–3888. 10.1523/JNEUROSCI.0936-06.2006 16611803 PMC6673885

[B101] Skaper S. D. (2007). The brain as a target for inflammatory processes and neuroprotective strategies. Ann. N.Y. Acad. Sci. 1122, 23–34. 10.1196/annals.1403.002 18077562

[B102] Subramaniam M. Henderson D. Campo P. Spongr V. (1992). The effect of “conditioning” on hearing loss from a high frequency traumatic exposure. Hear. Res. 58, 57–62. 10.1016/0378-5955(92)90008-B 1559906

[B103] Sun S. Yu H. Yu H. Honglin M. Ni W. Zhang Y.. (2015). Inhibition of the activation and recruitment of microglia-like cells protects against neomycin-induced ototoxicity. Mol. Neurobiol. 51, 252–267. 10.1007/s12035-014-8712-y 24781382

[B104] Syka J. Popelár J. (1980). Hearing threshold shifts from prolonged exposure to noise in guinea pigs. Hear. Res. 3, 205–213. 10.1016/0378-5955(80)90047-7 7440424

[B105] Tan W. J. Thorne P. R. Vlajkovic S. M. (2013). Noise-induced cochlear inflammation. World J. Otorhinolaryngol. 3:89 10.5319/wjo.v3.i3.89

[B106] Tan W. J. Thorne P. R. Vlajkovic S. M. (2016). Characterisation of cochlear inflammation in mice following acute and chronic noise exposure. Histochem. Cell Biol. 146, 219–230. 10.1007/s00418-016-1436-5 27109494

[B107] Tornabene S. V. Sato K. Pham L. Billings P. Keithley E. M. (2006). Immune cell recruitment following acoustic trauma. Hear. Res. 222, 115–124. 10.1016/j.heares.2006.09.004 17081714

[B108] Trowe M. O. Maier H. Schweizer M. Kispert A. (2008). Deafness in mice lacking the T-box transcription factor Tbx18 in otic fibrocytes. Development 135, 1725–1734. 10.1242/dev.014043 18353863

[B109] Vandesompele J. De Preter K. Pattyn F. Poppe B. Van Roy N. De Paepe A.. (2002). Accurate normalization of real-time quantitative RT-PCR data by geometric averaging of multiple internal control genes. Genome Biol. 3:RESEARCH0034. 10.1186/gb-2002-3-7-research0034 12184808 PMC126239

[B110] Vezzani A. Ravizza T. Balosso S. Aronica E. (2008). Glia as a source of cytokines: implications for neuronal excitability and survival. Epilepsia 49(Suppl. 2), 24–32. 10.1111/j.1528-1167.2008.01490.x 18226169

[B111] Viberg A. Canlon B. (2004). The guide to plotting a cochleogram. Hear. Res. 197, 1–10. 10.1016/j.heares.2004.04.016 15504598

[B112] Wang Y. Hirose K. Liberman M. C. (2002). Dynamics of noise-induced cellular injury and repair in the mouse cochlea. J. Assoc. Res. Otolaryngol. 3, 248–268. 10.1007/s101620020028 12382101 PMC3202415

[B113] Wang Z. Li H. (2000). Microglia-like cells in rat organ of Corti following aminoglycoside ototoxicity. Neuroreport 11, 1389–1393. 10.1097/00001756-200005150-00008 10841344

[B114] Worthington J. J. Fenton T. M. Czajkowska B. I. Klementowicz J. E. Travis M. A. (2012). Regulation of TGFβ in the immune system: an emerging role for integrins and dendritic cells. Immunobiology 217, 1259–1265. 10.1016/j.imbio.2012.06.009 22902140 PMC3690473

[B115] Xia A. Song Y. Wang R. Gao S. S. Clifton W. Raphael P.. (2013). Prestin regulation and function in residual outer hair cells after noise-induced hearing loss. PLoS ONE 8:e82602. 10.1371/journal.pone.0082602 24376553 PMC3869702

[B116] Yoshida K. Ichimiya I. Suzuki M. Mogi G. (1999). Effect of proinflammatory cytokines on cultured spiral ligament fibrocytes. Hear. Res. 137, 155–159. 10.1016/S0378-5955(99)00134-3 10545642

[B117] Zacharek M. A. Kaltenbach J. A. Mathog T. A. Zhang J. (2002). Effects of cochlear ablation on noise induced hyperactivity in the hamster dorsal cochlear nucleus: implications for the origin of noise induced tinnitus. Hear. Res. 172, 137–143. 10.1016/S0378-5955(02)00575-0 12361876

[B118] Zheng Y. Vagal S. McNamara E. Darlington C. L. Smith P. F. (2012). A dose–response analysis of the effects of L-baclofen on chronic tinnitus caused by acoustic trauma in rats. Neuropharmacology 62, 940–946. 10.1016/j.neuropharm.2011.09.027 22005094

