# Supplementary material for: The Role of Glia in the Peripheral and Central Auditory System Following Noise Overexposure: Contribution of TNF-α and IL-1β to the Pathogenesis of Hearing Loss
Source: Front Neuroanat. 2017 Feb 23;11:9. doi: 10.3389/fnana.2017.00009 (PMC5322242; doi:10.3389/fnana.2017.00009)
Supplement: Supplementary file 1 [file Table1.DOCX]

Supplementary Material

**The role of glia in the peripheral and central auditory system following noise overexposure: contribution of TNF-α and IL-1β to the pathogenesis of hearing loss**

Verónica Fuentes-Santamaría^1^ (*), Juan Carlos Alvarado^1^, Pedro Melgar-Rojas^1^, María Cruz Gabaldón-Ull^1^, Josef M. Miller^2,3,^ José M. Juiz^1^.

1. Instituto de Investigación en Discapacidades Neurológicas (IDINE), Albacete, Spain. Facultad de Medicina, Universidad de Castilla-La Mancha, Albacete, Spain.

2. Karolinska Institutet, Stockholm, Sweden.

3. University of Michigan, Ann Arbor, MI, USA.

(*) Correspondence to: Verónica Fuentes-Santamaria, PhD, Facultad de Medicina, Universidad de Castilla-La Mancha, Campus de Albacete. Calle Almansa 14, 02006, Albacete, Spain. Phone: (34) 967599200, ext 2933 Fax (34) 967599327.

E-mail address: Veronica. [Fuentes@uclm.es](mailto:Fuentes@uclm.es)

**Supplementary Table: 1**

**TABLE 1**

**Antibodies used for Immunohistochemistry**

| **Primary Antibody** | **Immunogen** | **Host** | **Code/clone** | **Dilution** | **Manufacturer** |
| --- | --- | --- | --- | --- | --- |
| Prestin | N-terminus of human prestin | Goat | SC-22692 | 1:100 | Santa Cruz, Biotechnology, Inc. Germany |
| Iba-1 | C-terminus of Iba1´  (N´-PTGPPAKKAISELP-C´) | Rabbit | 019-19741 | 1:2000 | Wako Pure Chemical Industries, Neuss, Germany |
| GFAP | Cow spinal cord GFAP | Rabbit | Z0334 | 1:2000 | Dako, Glostrup, Denmark |
| NeuN | Purified cell nuclei from mouse brain | Mouse | MAB337 | 1:200 | Millipore, Billerica, MA, USA |
| CR | Rat CR | Goat | AB1550 | 1:1000 | Millipore, Billerica, MA, USA |
| TNF-α | N-terminus of mouse TNF-α | Goat | SC-1351 | 1:100 | Santa Cruz, Biotechnology, Inc. Germany |
| IL-1β | C-terminus of rat IL-1β | Goat | SC-1252 | 1:100 | Santa Cruz, Biotechnology, Inc. Germany |
