# Supplementary material for: The Role of Glia in the Peripheral and Central Auditory System Following Noise Overexposure: Contribution of TNF-α and IL-1β to the Pathogenesis of Hearing Loss
Source: Front Neuroanat. 2017 Feb 23;11:9. doi: 10.3389/fnana.2017.00009 (PMC5322242; doi:10.3389/fnana.2017.00009)
Supplement: Supplementary file 2 [file Table2.DOCX]

Supplementary Material

**The role of glia in the peripheral and central auditory system following noise overexposure: contribution of TNF-α and IL-1β to the pathogenesis of hearing loss**

Verónica Fuentes-Santamaría^1^ (*), Juan Carlos Alvarado^1^, Pedro Melgar-Rojas^1^, María Cruz Gabaldón-Ull^1^, Josef M. Miller^2,3,^ José M. Juiz^1^.

1. Instituto de Investigación en Discapacidades Neurológicas (IDINE), Albacete, Spain. Facultad de Medicina, Universidad de Castilla-La Mancha, Albacete, Spain.

2. Karolinska Institutet, Stockholm, Sweden.

3. University of Michigan, Ann Arbor, MI, USA.

(*) Correspondence to: Verónica Fuentes-Santamaria, PhD, Facultad de Medicina, Universidad de Castilla-La Mancha, Campus de Albacete. Calle Almansa 14, 02006, Albacete, Spain. Phone: (34) 967599200, ext 2933 Fax (34) 967599327.

E-mail address: Veronica. [Fuentes@uclm.es](mailto:Fuentes@uclm.es)

**Supplementary Table: 2**

**TABLE 2**

### Oligonucleotides and qPCR parameters

| **Gene** | **GeneBank Accession No.** | **Primer sequence (5’–3’)** | **Genomic location (exons; FW** – **RV)** | **Product size (bp)** | **PCR efficiency** | **Regression coefficient (R^2^)** |
| --- | --- | --- | --- | --- | --- | --- |
| *Iba 1* | NM_017196.3 | FW: TGCTGAAAGCCCAACAGGAA  RV: CGTCTTGAAGGCCTCCAGTT | 3 – 3/4* | 113 | 90.8% | 0.9891 |
| *ICAM-1* | NM_012967.1 | FW: TCCTCCAATGGCTTCAACCC  RV: GGATGGATACCTGAGCACCG | 2 – 3/4 | 101 | 102.1% | 0.9987 |
| *IL1-β* | NM_031512.2 | FW: AGCTTTCGACAGTGAGGAGAA  RV: TCATCTGGACAGCCCAAGTC | 2/3 – 4 | 99 | 97.7% | 0.9984 |
| *iNOS* | NM_012611.3 | FW: CGCCTTCAACACCAAGGTTG  RV: TCAGAGTCTGCCCATTGCTG | 13/14 – 14/15 | 128 | 105.7% | 0.9616 |
| *TGF-β* | NM_021578.2 | FW: AGGAGACGGAATACAGGGCT  RV: ACGTTTGGGACTGATCCCATT | 3/4 – 4/5 | 100 | 92.1% | 1.0000 |
| *TNF-α* | NM_012675.3 | FW: GAGAAGTTCCCAAATGGGCT  RV: TTGCTACGACGTGGGCTACG | 1/2 – 3/4 | 109 | 102.6% | 0.9853 |
| *Timp-1* | NM_053819.1 | FW: CGCTAGAGCAGATACCACGA  RV: ACAGCTACAGGCTTTACTGGA | 1/2 – 3/4 | 97 | 92.1% | 0.9801 |

*Primer that match on an exon–exon junction.
