# Supplementary material for: The Role of Glia in the Peripheral and Central Auditory System Following Noise Overexposure: Contribution of TNF-α and IL-1β to the Pathogenesis of Hearing Loss
Source: Front Neuroanat. 2017 Feb 23;11:9. doi: 10.3389/fnana.2017.00009 (PMC5322242; doi:10.3389/fnana.2017.00009)
Supplement: Supplementary file 6 [file Table6.DOCX]

Supplementary Material

**The role of glia in the peripheral and central auditory system following noise overexposure: contribution of TNF-α and IL-1β to the pathogenesis of hearing loss**

Verónica Fuentes-Santamaría^1^ (*), Juan Carlos Alvarado^1^, Pedro Melgar-Rojas^1^, María Cruz Gabaldón-Ull^1^, Josef M. Miller^2,3,^ José M. Juiz^1^.

1. Instituto de Investigación en Discapacidades Neurológicas (IDINE), Albacete, Spain. Facultad de Medicina, Universidad de Castilla-La Mancha, Albacete, Spain.

2. Karolinska Institutet, Stockholm, Sweden.

3. University of Michigan, Ann Arbor, MI, USA.

(*) Correspondence to: Verónica Fuentes-Santamaria, PhD, Facultad de Medicina, Universidad de Castilla-La Mancha, Campus de Albacete. Calle Almansa 14, 02006, Albacete, Spain. Phone: (34) 967599200, ext 2933 Fax (34) 967599327.

E-mail address: Veronica. [Fuentes@uclm.es](mailto:Fuentes@uclm.es)

**Supplementary Table: 6**

**TABLE 6**

### mRNA expression levels of inflammation-related genes in control and noise-exposed animals

|  |  |  |  |  |  |  |  |  |
| --- | --- | --- | --- | --- | --- | --- | --- | --- |
| **Survival times** |  | Iba1 | ICAM-1 | IL-1β | iNOS | TGF-β | TNF-α | Timp1 |
| **Control (1)** |  | 0.97 ± 0.03 | 1.01 ± 0.04 | 1.01 ± 0.03 | 1.02 ± 0.04 | 1.02 ± 0.04 | 1.04 ± 0.04 | 1.02 ± 0.04 |
| **1d (2)** |  | 1.38 ± 0.05 | 1.42 ± 0.05 | 1.34 ± 0.05 | 1.47 ± 0.08 | 1.33 ± 0.06 | 1.42 ± 0.08 | 3.43 ± 0.33 |
| **10d (3)** |  | 1.35 ± 0.08 | 1.23 ± 0.06 | 1.57 ± 0.06 | 1.29 ± 0.12 | 1.21 ± 0.10 | 1.61 ± 0.12 | 1.49 ± 0.09 |
| **30d (4)** |  | 1.13 ± 0.04 | 1.15 ± 0.05 | 1.05 ± 0.06 | 1.19 ± 0.08 | 1.09 ± 0.04 | 1.33 ± 0.07 | 1.64 ± 0.06 |
|  |  |  |  |  |  |  |  |  |
| **Statistical Comparison** |  | **Significance levels** | | | | | | |
| 1 vs. 2 |  | *** | *** | *** | *** | *** | ** | *** |
| 1 vs. 3 |  | *** | * | *** | * | NS | *** | * |
| 1 vs. 4 |  | * | NS | NS | NS | NS | ** | ** |
| 2 vs. 3 |  | NS | NS | * | NS | NS | NS | *** |
| 2 vs. 4 |  | ** | * | ** | NS | * | NS | *** |
| 3 vs. 4 |  | ** | NS | ** | NS | NS | NS | NS |

Values are means ± standard errors. * p<0.05; ** p<0.01; *** p<0.001; NS, No significant.
