# Supplementary material for: The Role of Glia in the Peripheral and Central Auditory System Following Noise Overexposure: Contribution of TNF-α and IL-1β to the Pathogenesis of Hearing Loss
Source: Front Neuroanat. 2017 Feb 23;11:9. doi: 10.3389/fnana.2017.00009 (PMC5322242; doi:10.3389/fnana.2017.00009)
Supplement: Supplementary file 7 [file Table7.DOCX]

Supplementary Material

**The role of glia in the peripheral and central auditory system following noise overexposure: contribution of TNF-α and IL-1β to the pathogenesis of hearing loss**

Verónica Fuentes-Santamaría^1^ (*), Juan Carlos Alvarado^1^, Pedro Melgar-Rojas^1^, María Cruz Gabaldón-Ull^1^, Josef M. Miller^2,3,^ José M. Juiz^1^.

1. Instituto de Investigación en Discapacidades Neurológicas (IDINE), Albacete, Spain. Facultad de Medicina, Universidad de Castilla-La Mancha, Albacete, Spain.

2. Karolinska Institutet, Stockholm, Sweden.

3. University of Michigan, Ann Arbor, MI, USA.

(*) Correspondence to: Verónica Fuentes-Santamaria, PhD, Facultad de Medicina, Universidad de Castilla-La Mancha, Campus de Albacete. Calle Almansa 14, 02006, Albacete, Spain. Phone: (34) 967599200, ext 2933 Fax (34) 967599327.

E-mail address: Veronica. [Fuentes@uclm.es](mailto:Fuentes@uclm.es)

**Supplementary Table: 7**

**TABLE 7**

### Iba1 and GFAP immunostaining in the AVCN in control and noise-exposed animals

|  |  | **Mean gray levels** | |
| --- | --- | --- | --- |
| **Survival times** |  | **Iba1** | **GFAP** |
| **Control (1)** |  | 92.86 ± 2.77 | 97.97 ± 5.55 |
| **1d (2)** |  | 119.06 ± 2.05 | 121.63 ± 5.82 |
| **10d (3)** |  | 130.95 ± 1.99 | 123.74 ± 3.03 |
| **30d (4)** |  | 135.96 ± 2.16 | 127.19 ± 4.72 |
|  |  |  |  |
| **Statistical Comparison** |  | **Significance levels** | |
| 1 vs. 2 |  | *** | * |
| 1 vs. 3 |  | *** | * |
| 1 vs. 4 |  | *** | ** |
| 2 vs. 3 |  | ** | NS |
| 2 vs. 4 |  | *** | NS |
| 3 vs. 4 |  | ** | NS |

Values are means ± standard errors. * p<0.05; ** p<0.01; *** p<0.001;

NS, No significant
